# Genomic analysis reveals the biotechnological and industrial potential of levan producing halophilic extremophile, *Halomonas smyrnensis* AAD6T

**DOI:** 10.1186/s40064-015-1184-3

**Published:** 2015-08-04

**Authors:** Elif Diken, Tugba Ozer, Muzaffer Arikan, Zeliha Emrence, Ebru Toksoy Oner, Duran Ustek, Kazim Yalcin Arga

**Affiliations:** Department of Bioengineering, Marmara University, Goztepe, 34722 Istanbul, Turkey; Department of Genetics, Institute for Experimental Medicine, Istanbul University, Capa, 34093 Istanbul, Turkey; Department of Medical Genetics, School of Medicine, REMER, Medipol University, 34810 Istanbul, Turkey

**Keywords:** *Halomonas smyrnensis*, Halophiles, Genome, Next-generation sequencing, Levan

## Abstract

*Halomonas smyrnensis* AAD6T is a gram negative, aerobic, and moderately halophilic bacterium, and is known to produce high levels of levan with many potential uses in foods, feeds, cosmetics, pharmaceutical and chemical industries due to its outstanding properties. Here, the whole-genome analysis was performed to gain more insight about the biological mechanisms, and the whole-genome organization of the bacterium. Industrially crucial genes, including the levansucrase, were detected and the genome-scale metabolic model of *H*. *smyrnensis* AAD6T was reconstructed. The bacterium was found to have many potential applications in biotechnology not only being a levan producer, but also because of its capacity to produce Pel exopolysaccharide, polyhydroxyalkanoates, and osmoprotectants. The genomic information presented here will not only provide additional information to enhance our understanding of the genetic and metabolic network of halophilic bacteria, but also accelerate the research on systematical design of engineering strategies for biotechnology applications.

**Electronic supplementary material:**

The online version of this article (doi:10.1186/s40064-015-1184-3) contains supplementary material, which is available to authorized users.

## Background

*Halomonas smyrnensis* AAD6T is a rod-shaped, Gram-negative, aerobic, exopolysaccharide (EPS) producing and moderately halophilic bacterium, growing at salt concentration in the range of 3–25% (w/v) NaCl (optimum, 10%), at temperatures between 5 and 40°C (optimum, 37°C), and pH values between 5.5 and 8.5 (optimum, 7.0) (Poli et al. 2012). The strain utilizes glucose, sucrose, maltose, arabinose, raffinose, fructose, galactose, and mannose as a sole carbon and energy source, and alanine and serine as a sole carbon, nitrogen and energy source (Poli et al. 2009, 2012). The EPS production by *H*. *smyrnensis* AAD6T is growth-associated, and when sucrose was used as carbon source the bacteria excrete high levels of levan, which is a long linear homopolymeric EPS of ß(2-6)-linked fructose residues (Poli et al. 2009). Levan has been credited as one of the most promising polysaccharides for foods, feeds, cosmetics, pharmaceutical and chemical industries (Donot et al. 2012) and, potential uses of levan produced by AAD6T, as a bioactive polymer, were reported (Costa et al. 2013; Kucukasik et al. 2011; Sam et al. 2011; Sezer et al. 2011; Sima et al. 2011, 2012).

When compared to the other groups of extremophilic microorganisms such as the thermophiles, the halophiles have been relatively little exploited in biotechnological processes with a few exceptions (Pastor et al. 2010; Philip et al. 2007; Rodriguez-Valera and Lillo 1992; Sam et al. 2011). On the other hand, due to their osmoadaptation abilities and metabolic capabilities for the production of compatible solutes, bioplastics, nutritional products, biopolymers, and halophilic enzymes, they represent considerable potential in various industries including chemical, environmental, biofuel, medical, pharmaceutical and health care.

In systems biology research, the microbial genome sequence is the starting point for detailed analysis of identifying gene-protein associations and metabolic reconstruction. In this context, next generation sequencing (NGS) technologies play an important role since they provide high-throughput genomic data, including whole genome sequences (WGS), at unprecedented speed with relatively low cost (Gov and Arga 2014; Zhang et al. 2011). WGS data provide many advantages in comparative genomics and metabolic engineering. In addition to the nucleotide sequence and protein sequences of the encoded proteins, the gene content, the order of genes and the predicted enzymes on the genome may be informative for phylogenetic analyses and metabolic reconstructions. Moreover, WGS information facilitates the acquirement of functional genomics data.

To design economical production schemes, fermentation conditions and effective stimulatory factors for levan production by *H*. *smyrnensis* AAD6T was analyzed systematically (Ateş et al. 2013; Ates et al. 2011; Sarilmiser Kazak et al. 2014). However, towards the design of efficient microbial cell factories for levan overproduction, the main bottleneck was the lack of information on the genome of the microorganism. Considering this fact, we employed two different NGS technologies, namely, Roche 454 GS FLX+ System and Ion Torrent Sequencer, and a hybrid NGS approach to develop a high-quality WGS data for *H*. *smyrnensis* AAD6T (Sogutcu et al. 2012). Within the context of this study, the genome of AAD6T was further analyzed to reveal the essential biological mechanisms and the whole genomic organization of levan producing *H*. *smyrnensis* AAD6T, and the genome-scale metabolic model of *H*. *smyrnensis* AAD6T was reconstructed. This understanding is crucial for rational design and optimization of engineering strategies for levan overproduction. Furthermore, the information provided here will facilitate future studies on the genetic and metabolic diversity of halophilic bacteria and contribute to the employment of *H*. *smyrnensis* AAD6 in biotechnological processes.

## Methods

### Bacterial strain

Halophilic bacterial strain *H*. *smyrnensis* AAD6T (JCM 15723, DSM 21644) used in this study was isolated from Çamaltı Saltern Area in Turkey (Poli et al. 2012).

### Sequencing and assembly

The genomic DNA was isolated from exponential-phase cultures using the quick protocol of Wizard^®^ Genomic DNA Purification Kit (Promega, Madison, WI). The optimum semi-chemical medium (Poli et al. 2009) was used in growth cultures. To obtain the highest growth rate without EPS production, glucose was added as sole carbon source to chemical medium at concentration of 10 g/L.

The genome of *H*. *smyrnensis* AAD6T was sequenced by the whole genome shotgun approach using a combination of 454 GS FLX+ (454 Life Sciences, Branford, CT) and Ion Torrent (Ion Torrent Systems, Inc., Guilford, CT) sequencers and the generated shotgun sequence data was assembled as described in (Sogutcu et al. 2012).

### Genome annotation

Gene prediction and genome annotation were carried out using the RAST auto-annotation server (Aziz et al. 2008). Protein-encoding and transfer RNA genes were predicted by RAST server, while the ribosomal RNA genes were identified with RNAmmer (v1.2) (Lagesen et al. 2007). The gene predictions of essential biosystems were manually verified using BLAST searches against protein databases, the Universal Protein Resource (UniProt) (http://www.uniprot.org/) and National Center for Biotechnology Information (NCBI) (http://www.ncbi.nlm.nih.gov/). The gene functions and classifications were based on the subsystem annotation of the RAST server. Information on enzyme-encoding genes was taken from Kyoto Encyclopedia of Genes and Genomes (KEGG) (Kanehisa et al. 2012) and ExPaSy databases (Artimo et al. 2012). Transport protein coding genes were annotated using the similarity searches against the Transfer Classification Database (TCDB) (Saier et al. 2009).

### Phylogenetic and phylogenomic analyses

The phylogenetic analyses were performed using PhyML (v3.0) and phylogenetic trees were reconstructed using maximum likelihood method (Guindon et al. 2010). The phylogenetic classification of bacterial strains was based on complete 16S rRNA sequences. The phylogenetic classification of a set of bacterial levansucrase enzymes was based on nucleotide sequences taken from NCBI database. The phylogenomic comparison within the *Halomonadaceae* family was based on the encoded proteins with putative functions assigned by the RAST server. Encoded proteins were sorted out for all genomes and comparatively analyzed based on the presence/absence of proteins.

### Reconstruction of the genome-scale metabolic model

The entire reconstruction procedure consists of an automated procedure followed by manual curation at the gap filing step. The preliminary model was built automatically via the Model SEED (Henry et al. 2010). The refinement of the model (for stand-alone reactions or metabolites, and missing connections in essential metabolic processes) was achieved via a non-automated but iterative decision-making process, as described in (Ates et al. 2011).

## Results and discussion

### Sequencing and developing the *H*. *smyrnensis* AAD6T genome

**Fig. 1:**
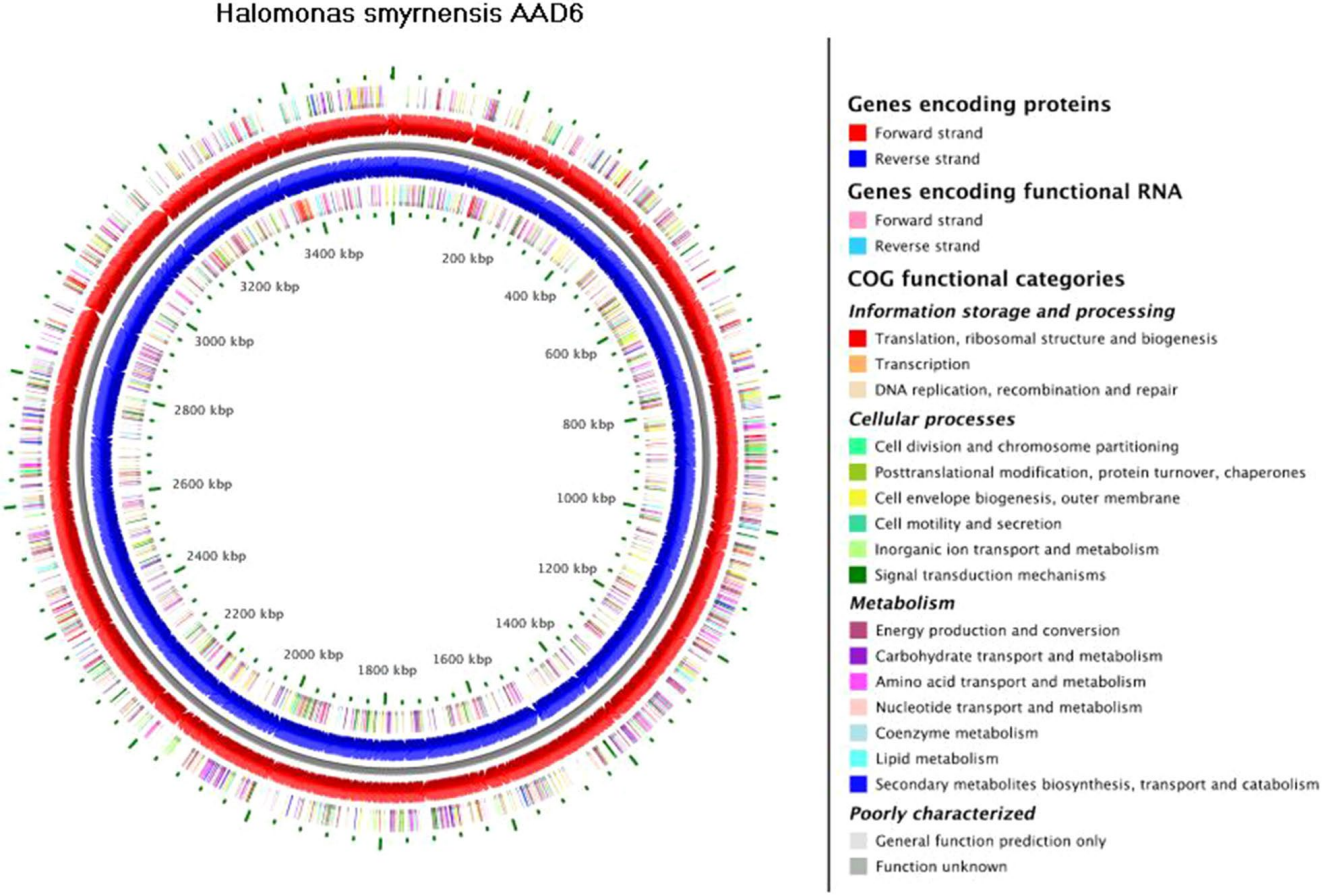
*Circular* representation of draft genome sequence of *H*. *smyrnensis* AAD6T. The *inner* and *outer scales* designate the coordinates in two hundreds kilo base pair increments. The *red* and *blue circles* show the predicted coding regions of AAD6 on the forward and reverse strands, respectively. The clustering of putative genes on the forward and reverse strands to Clusters of Orthologous Group (COG) functional categories was represented by *differential colors* in the *outer* and *inner circles*, respectively.

The WGS project of *H*. *smyrnensis* AAD6T (Sogutcu et al. 2012) was comprised of data from Roche 454 GS FLX+ platform and Ion Torrent sequencer (Table 1). GS FLX+ platform provided longer reads (with mean length of 499.46 bp) and higher coverage (76-fold in average), when compared to Ion Torrent System (with mean length of 113.01 bp and 11-fold coverage). On the other hand, Ion Torrent System represented a duplication rate of 0.09%, which is significantly lower than that of GS FLX+ platform, i.e. 9.47% in average. Consequently, the de novo assembly procedure resulted in a total assembly size of 3.56 Mbp and is presumed to be a single circular chromosome (Fig. 1).

**Table 1:** High-throughput sequencing statistics

Sequencing method	Total bases (Mbp)	Total reads	Duplicate (%)	Reads used^a^	Mean read length (bp)	Longest read (bp)	Theoretical coverage^b^
GS FLX+ (R1)	307.39	556,192	9.01	506,091	552.67	1,180	86.3
GS FLX+ (R2)	233.31	526,372	9.96	473,924	443.24	1,340	65.5
GS FLX+ (Total)	540.70	1,082,564	9.47	980,015	499.46	1,340	151.8
Ion Torrent (IT)	40.67	359,877	0.09	359,558	113.01	222	11.4
Overall	581.37	1,442,441	7.13	1,339,573	403.05	1,340	163.3

^a^After removal of duplicates.

^b^Based on draft genome size of 3.56 Mbp.

### General features of the *H*. *smyrnensis* AAD6T genome

**Fig. 2:**
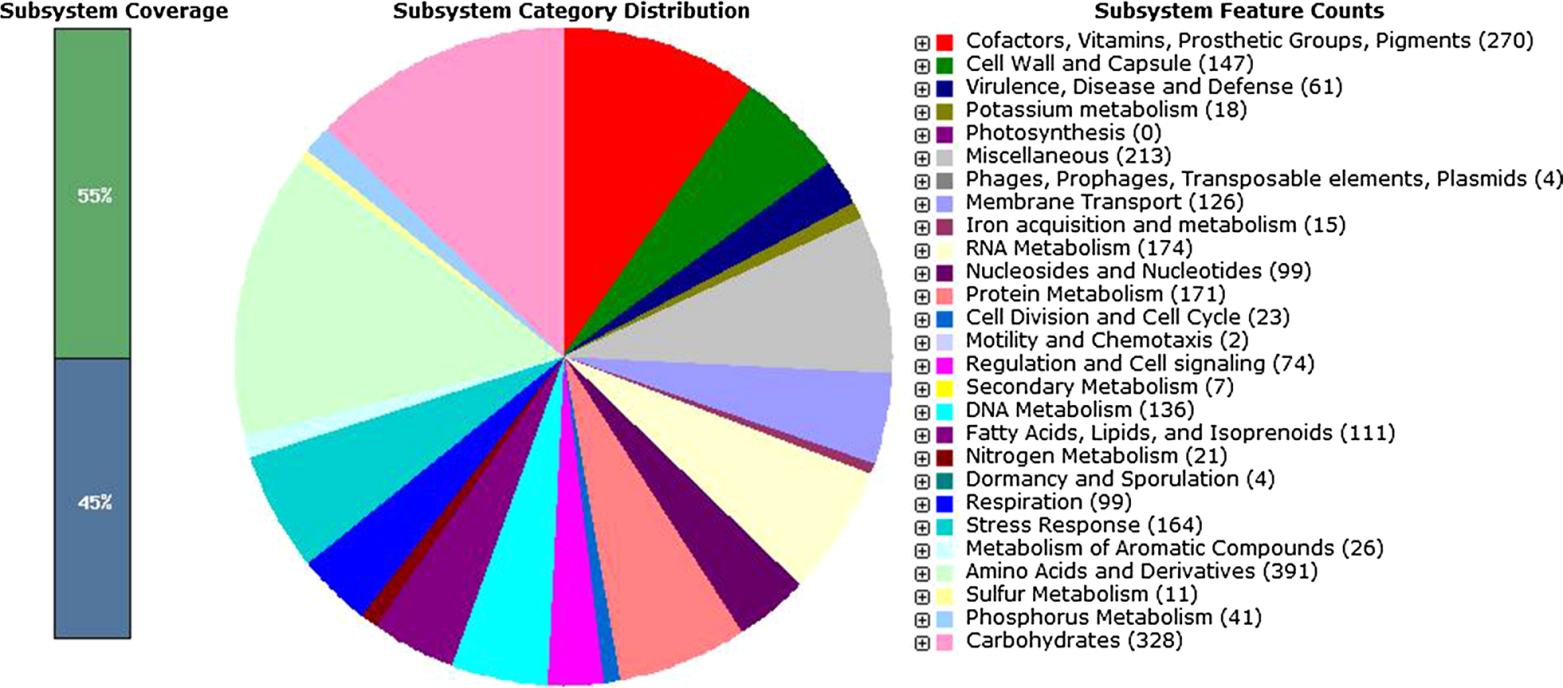
Subsystem distribution of coding sequences in the draft genome of *H*. *smyrnensis* AAD6T. The *bar * on the *left* indicates the clustered (*green*) and unclustered (*blue*) percentages of coding sequences. Distribution of coding sequences into subsystems is shown in *pie graph*. The *legend on the right* designates the *color* associated with each subsystem.

The gene prediction and genome annotation of draft genome of *H*. *smyrnensis* AAD6T resulted in a total of 3,237 coding sequences and 64 RNA genes (Table 2, Additional files 1, 2). Putative functions could be assigned to 2,436 protein-coding genes, whereas 801 hypothetical proteins had no match to any known proteins. The major part (55%) of the coding sequences was clustered in 449 subsystems (Additional file 3); however 1,481 coding sequences were not included in any subsystem. With regard to the subsystem distribution of coding sequences (Fig. 2), the largest number of these sequences were found in “Amino acids and Derivatives” and “Carbohydrates” categories with 391 and 328 coding sequences, respectively. *H*. *smyrnensis* AAD6T has significantly more coding sequences related to “osmotic stress” subsystem under “Stress Response” category (56 coding sequences) when compared to *Escherichia coli* K12 and *Bacillus cereus* AH187 (22 and 13 coding sequences, respectively). Most of the osmotic stress genes of *H*. *smyrnensis* AAD6T (47 coding sequences) encode proteins associated with choline and glycine betaine uptake, and glycine betaine biosynthesis pathways, which are involved in osmoprotection.

**Table 2:** General features of the draft genome of *H*. *smyrnensis* AAD6

	Genome features
Genome	*H*. *smyrnensis* AAD6
Domain	Bacteria
Taxonomy	Proteobacteria; Gammaproteobacteria; Halomonadaceae; Halomonas
Size	3,561,919 bp
G+C content	67.9%
Number of subsystems	449
Open reading frames	
Number of coding sequence	3,237
Coding regions length	3,095,766 bp
Percentage coding	86.9%
Number of RNAs	
16S	1
23S	1
5S	1
Transfer RNAs	61

At a large-scale comparison, the size of the genome, the number of coding regions and the coding capacity of AAD6T were slightly smaller than those of other halophilic bacteria whose genome has already been sequenced (Table 3). On the other hand, the GC content of *H*. *smyrnensis* AAD6T seems to be slightly higher than that of *H*. *elongata* DSM 2581 (Schwibbert et al. 2011) and *C*. *salexigens* DSM 3043 (Copeland et al. 2011), but the GC contents of genomes of halophilic bacteria vary in a large range from 35.7% (*Oceanobacillus iheyensis* HTE831) (Takami et al. 2002) to 74.3% (*Halomonas ventosae* sp. nov.) (Martínez-Cánovas et al. 2004).

**Table 3:** Comparison of genome characteristics of halophilic bacteria

**Isolate**	*H*. *smyrnensis* AAD6 (DSM 21644)	*H*. *elongata* DSM 2581	*C*. *salexigens* DSM 3043
Genome size (Mb)	3.56	4.06	3.69
GC (%)	67.9	63.6	63.9
Coding regions	3,237	3,691	3,341
Avg. length of coding sequences^a^	956.4	949.3	959.4
Coding capacity (%)	86.9	88.1	88.9

^a^Length of total coding regions/number of coding regions.

### Phylogenetic and phylogenomic relatedness to other microorganisms

Phylogenetic classification based on 16S rRNA sequences places *H*. *smyrnensis* AAD6T in the *Halomonas* genus of the *Halomonadaceae* family (Poli et al. 2012). *H*. *salina* F8-11 (99.5%) and *H*. *halophila* CCM 3662 (99.5%) were found to be the closest species based on 16S rRNA gene sequence comparison (Additional file 4: Figure S1a). On the other hand, based on the phylogenetic classification of bacterial strains whose WGS data were available (comprising of 2 extreme halophilic, 10 moderate halophilic, 15 mesophilic, and 29 non-halophilic bacterial strains), two halophilic bacteria, *H*. *elongata* DSM 2581 and *C*. *salexigens* DSM 3043, were the most closely related organisms to *H*. *smyrnensis* AAD6T (Additional file 4: Figure S1b).

Rapid Annotation using Subsystem Technology (RAST) server establishes the phylogenetic context with using representative sequences such as tRNA synthetases, which are admitted as universal or nearly universal in prokaryotes (Aziz et al. 2008). The phylogenetic comparison of *H*. *smyrnensis* AAD6T genome based on these representative sequences with genomes available pointed out the closest similarity with *C*. *salexigens* DSM 3043 (with a score of 524), followed by *H*. *elongata* DSM 2581 (with the score of 346). Transfer RNAs (tRNAs) are thought to be among the oldest and highly conserved sequences on earth, and are often involved in horizontal gene transfer (Widmann et al. 2010). The partnership of horizontal genes between *H*. *smyrnensis* AAD6T and *C*. *salexigens* DSM 3043 may cause such a different phylogenetic relatedness compared to 16S rRNA based analysis.

**Fig. 3:**
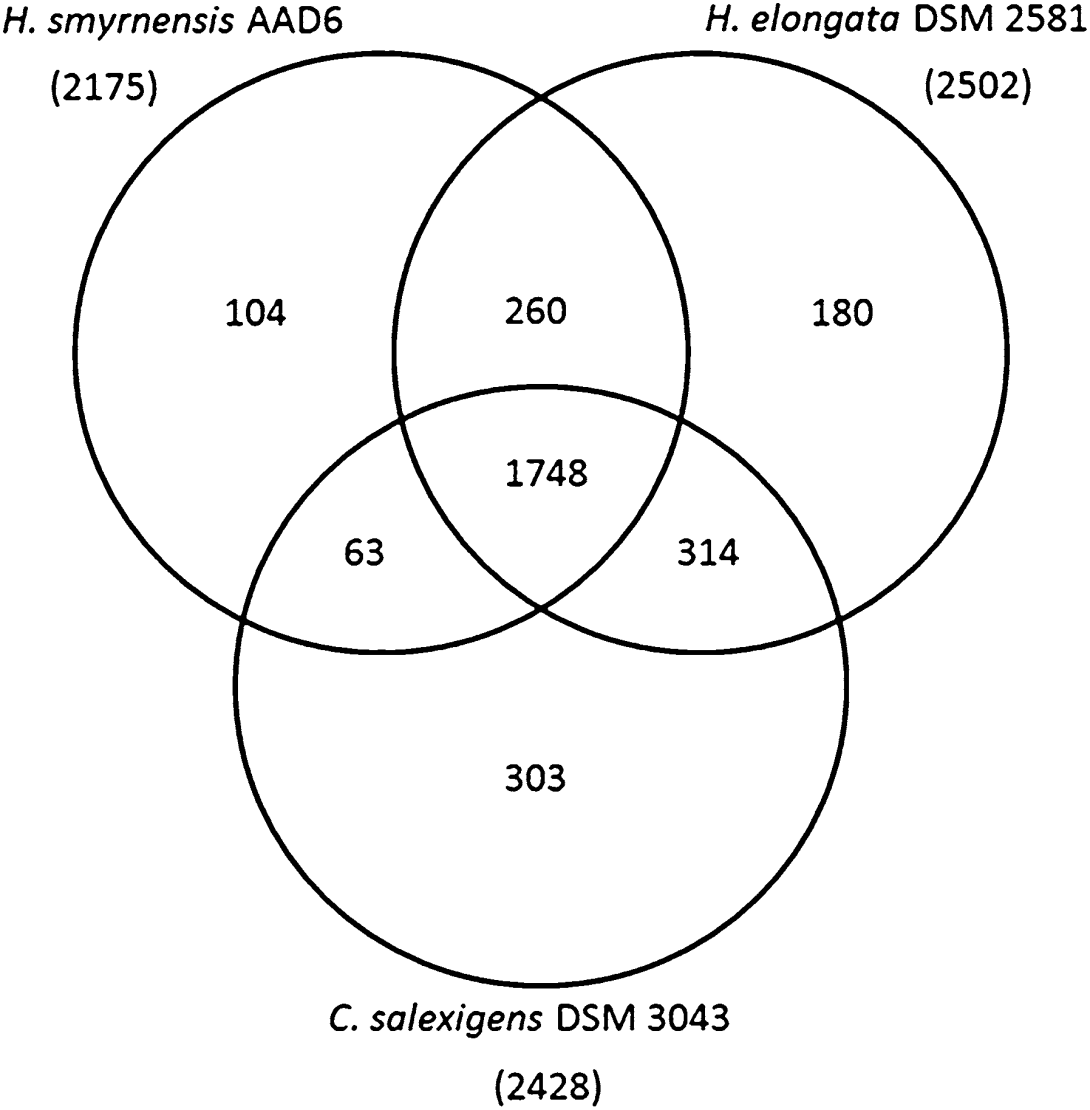
*Venn* diagram comparing the encoded proteins of three halophilic bacteria. Total numbers of proteins encoded in the genomes are given in *parenthesis*. The numbers of shared and unique proteins are shown inside the diagram.

The close relationships between these three moderately halophilic strains from *Halomonadaceae* family were also confirmed with the phylogenomic analysis within three genomes, which was based on the encoded proteins with putative function assignment (Fig. 3). Accordingly, 92.3% of the total predicted proteins in *H*. *smyrnensis* AAD6T were encoded by *H*. *elongata* DSM 2581; while 1,811 proteins that were 83.3% of the total predicted proteins in *H*. *smyrnensis* AAD6T were also encoded by *C*. *salexigens* DSM 3043. In addition, 80.4% of the total proteins encoded by the *H*. *smyrnensis* AAD6T (1,748 proteins) were also encoded by the genomes of all three strains. This finding indicates that at least 80% of the *H*. *smyrnensis* AAD6T genome has been vertically transmitted from a common ancestor of the *Halomonadaceae* family, comprising the backbone of the *H*. *smyrnensis* AAD6T genome. The rest of the genome encodes proteins similar to those produced by bacterial species other than those in the *Halomonadaceae* family. A large fraction of the 1,748 core proteins (Fig. 3) was assigned to categories with general cellular processes such as energy production and conversion, amino acid transport and metabolism, central carbon metabolism, translation, and transcription. On the other hand, 104 proteins from diverse categories were unique to *H*. *smyrnensis* AAD6T. Among these categories, Arginine biosynthesis with ten enzymes (EC 1.2.1.38, EC 2.1.3.3, EC 2.3.1.1, EC 2.3.1.35, EC 2.6.1.11, EC 2.7.2.8, EC 3.5.1.16, EC 3.5.1.18, EC 4.3.2.1, EC 6.3.4.5) and a regulatory protein (ArgR), and auxin biosynthesis with 4 proteins (EC 2.4.2.18, EC 5.3.1.24, alpha and beta chains of EC 4.2.1.20) drew the attention.

### Metabolism

To facilitate reconstruction of genome scale metabolic network of AAD6T, metabolic proteins and enzymes were identified with the open frame shifts that are encoded these proteins and enzymes via whole genome annotation (Additional file 5). The gene annotations revealed that the complete pathways of glycolysis (Embden–Meyerhof–Parnas, EMP), Entner–Doudoroff (ED), gluconeogenesis, pentose-phosphate and all de novo amino acid biosynthesis were represented in the *H*. *smyrnensis* AAD6T genome. The TCA cycle and glyoxylate shunt were also complete and genes corresponding to aerobic respiration pathways were detected. The biosynthetic pathways for most vitamins were found; however, pathways for riboflavin (vitamin B2) and pyrroloquinoline quinone syntheses were notably absent.

An important point to be discussed is the pathway utilized in glucose degradation in halophilic bacteria. Although complete EMP pathway was annotated in the genome of *C*. *salexigens*, glucose was degraded via the ED pathway (Pastor et al. 2013). Performing a bioinformatics analysis on phosphofructokinases (PFKs), it was shown that the PFKs from *C*. *salexigens* (Csal_1534) and *H*. *elongata* (Helo_2186) are only remotely similar to functional characterized PFKs. As a result, they hypothesized that the Csal_1534 protein functions as fructose-bisphosphatase, rather than as PFK. The genome annotation of *H*. *smyrnensis* AAD6T presented Peg.1123 as PFK and also failed to identify the presence of any fructose-bisphosphatase. However, PFK of *H*. *smyrnensis* AAD6T displays significant sequence similarity with the PFKs from *C*. *salexigens* (Csal_1534) and *H*. *elongata* (Helo_2186), raising the possibility that Peg.1123 may also be a fructose-biphosphatase. It is therefore doubtful whether *H*. *smyrnensis* AAD6T has a functional EMP pathway. On the other hand, on the basis of the genome annotation of *H*. *smyrnensis* AAD6T, one of the three variants of the ED pathway, the phosphorylative ED pathway from glucose or d-gluconate to 6-phosphogluconate, could be reconstructed, as in the case of *C*. *salexigens* (Pastor et al. 2013).

The genome annotation results for *H*. *smyrnensis* AAD6T include information required to reconstruct the stoichiometric metabolic model of *H*. *smyrnensis* AAD6T, which reflects the metabolic capabilities of the bacterium. The metabolic model was reconstructed systematically via automatic building followed by manual curation at the gap filling step (Additional file 6). The resulting *i*KYA1142 metabolic model comprises 980 metabolites and 1,142 metabolic reactions. A total of 819 protein-encoding genes were included in the metabolic model, which represents approximately 33.5% of the genes with assigned function in the *H*. *smyrnensis* AAD6T genome.

As a consequence of validation and constraints-based flux analyses, the genome-scale reconstructed metabolic model of *H*. *smyrnensis* AAD6T, *i*KYA1142, is expected to be useful for medium design and the development of metabolic engineering strategies for increased levan production, and systematical design of strain development strategies for biotechnology applications. Consequently, the reconstructed model together with the whole genome information presented here will be a crucial pathfinder towards future in silico and in vivo studies of this organism.

### Transport

Transport systems are vitally important for all organisms and also play a key role in halophiles for adaptation to high salinity environments. In the *H*. *smyrnensis* AAD6T genome, 307 open reading frames (ORFs) (9.5% of total) were found to be involved in various transport systems which enable the bacteria to accumulate needed nutrients, extrude unwanted by-products and maintain cytoplasmic content of protons and salts conducive to growth and development. This proportion does not show a major difference in other halophilic and non-halophilic microorganisms taken into comparison (Blattner et al. 1997; Copeland et al. 2011; Kunst et al. 1997; Pastor et al. 2013; Schwibbert et al. 2011).

Major part of transporter genes in *H*. *smyrnensis* AAD6T genome belongs to Tripartite ATP-independent periplasmic (TRAP) transporters that are a large family of solute transporters ubiquitous in bacteria and archaea, but absent in eukaryotes, however the number of TRAP transporter genes in *E*. *coli* (Blattner et al. 1997) genome is much lower compared to *H*. *smyrnensis* AAD6T genome.

At the same time, halophilic genomes exhibit significant differences in ion transport systems. For instance, *H*. *smyrnensis* AAD6T was shown specific requirement to presence of Na^+^ and Cl^−^ ions, in absence of these ions the strain does not grow (Poli et al. 2009). Accordingly, its genome has significantly more (i.e., ten genes) Na^+^ and Cl^−^ transporter genes like other halophilic bacteria [Eight genes in *C*. *salexigens* (Copeland et al. 2011) and nine genes in *H*. *elongata* DSM 2581 (Schwibbert et al. 2011)] than non-halophilic bacteria [two genes in *E*. *coli* K12 (Blattner et al. 1997) and three genes in *B*. *subtilis* (Kunst et al. 1997)]. This larger Na^+^ transporter portion may provide more Na^+^ gradients to drive transport processes in the cell membrane (Ventosa et al. 1998). Among the ORFs, 183 were categorized under subsystems whereas 124 were identified as putative transporters. They include transporters for sugars and carbohydrates (maltose/maltodextrin, inositol, d-xylose, succinoglycan, mannitol), ions (Na^+^, K^+^, Mg^2+^, Zn^2+^, Mn^2+^, Fe^2+^/^3+^, Co^2+^, Ni^2+^ and Mo^2+^), anions and cations (sulphate, ammonium, nitrate, nitrite, phosphate, phosphonates, sulfonates, and malonate), amino acids (arginine, ornithine, histidine, methionine, glutamic acid, glutamine, alanine, glycine, aspartate and threonine), and other molecules (cobalamin, thiamine, urea, C4 dicarboxylates, uracil, xanthine, auxin, spermidine/putrescine, glycine betaine, l-proline, benzoate, *N*-acetylneuraminate, glycerol-3-phosphate, l-lactate, cyclase, oligoketide and various polyols). In addition, utilization systems were present for glucose, fructose, galactose, sucrose, arabinose, mannose and rhamnose.

### Osmoregulation

Osmolarity resistance (or tolerance) is one of the most important characteristics of *H*. *smyrnensis* AAD6T, since the microorganism can grow in media containing up to 3.4 mol/L sodium chloride (Poli et al. 2012). Accumulating intracellular organic solutes, such as ectoine and betaine, and rapidly releasing those solutes when extracellular osmolarity declines, is a frequently observed osmolarity resistance strategy when extracellular osmolarity rises (Oren 2002). Here, using genome annotation predictions, pathways associated with uptake of choline-betaine, biosynthesis of glycine betaine (GB), ectoine and hydroxyectoine were reported for *H*. *smyrnensis* AAD6T.

*Halomonassmyrnensis* AAD6T possesses two different GB/proline betaine (PB) high-affinity uptake systems, OpuA, known from *B*. *subtilis* (Kempf et al. 1997) and ProU, known from *E*. *coli* (Schiefner et al. 2004). The OpuA system is a member of ABC transporter superfamily and consists of a membrane-associated ATPase (OpuAA), the integral membrane protein (OpuAB), and the extracellular ligand-binding protein (OpuAC). The extracellular OpuAC protein binds GB or PB with high affinity, and delivers it to the OpuAA/OpuAB protein complex for the release of substrate into the cytosol with an ATP-dependent substrate translocation mechanism. *H*. *smyrnensis* AAD6T also has a second ABC transporter system ProU for GB/PB import into the cytoplasm. ProU transport system is composed of a GB/PB binding protein ProX and permease protein ProW and ProV. The ProX protein is binding to GB/PB for the substrate translocation via ProWV permease protein complex into the cytosol. GB can also be synthesized from the precursor choline. In this sense, annotations also included a high-affinity choline uptake protein BetT and two enzymes: choline dehydrogenase (EC 1.1.99.1) that catalyzes the conversion of choline into betaine aldehyde as the intermediate and betaine aldehyde dehyrogenase (EC 1.2.1.8) that catalyzes the conversion of betaine aldehyde to GB (Additional file 4: Figure S2). Choline and GB may function as stress protectants, not only against osmotic stress but also against high-temperature and low-temperature challenges (Hoffmann and Bremer 2011).

Ectoine and hydroxyectoine are widely distributed compatible solutes accumulated by halophilic and halotolerant microorganisms to prevent osmotic stress in highly saline environments. Ectoine presents industrial importance with current and potential applications as protecting agents for macromolecules, cells and tissues, together with its potential as therapeutic agents for certain diseases (Pastor et al. 2010). Genome analysis revealed three genes (Peg.2770, Peg.2771, and Peg.2772), which were located together on the chromosome of *H*. *smyrnensis* AAD6T, encoding enzymes of ectoine biosynthesis pathway, i.e. EctA (l-2,4-diaminobutyric acid acetyltransferase, EC 2.3.1.-), EctB (diaminobutyrate-pyruvate aminotransferase, EC 2.6.1.46), and EctC (l-ectoine synthase, EC 4.2.1.-,), respectively. In addition, the ectD gene (peg.3147) encoding ectoine hydroxylase (EC 1.17.-.-), which is involved in the conversion of ectoine to hydroxyectoine, is also present. On the other hand, any gene encoding neither a transcriptional regulator nor an aspartokinase specific for ectoine biosynthesis could not found adjacent to ectABC genes. Unlike *C*. *salexigens* and *H*. *elongata* genomes, the genome of *H*. *smyrnensis* AAD6T does not possess any genes for the degradation of ectoine.

### Levan exopolysaccharide biosynthesis

Genome analysis revealed *Hs_SacB* gene encoding the extracellular levansucrase enzyme (EC 2.4.1.10) which catalyzes levan synthesis from sucrose-based substrates by transfructosylation (Donot et al. 2012). The levansucrase belongs to the family of glycosyltransferases, specifically the hexosyltransferases. The length of the *Hs_SacB* gene was 1,251 bp and it is predicted to encode a protein of 416 amino acids. The amino acid distribution in *Hs_SacB* indicates the dominance of hydrophobic amino acids (with 45.2%), whereas polar amino acids and charged amino acids constitute 32.2 and 22.5%, respectively (Additional file 4: Figure S3). The high frequency of glycine (G) and proline (P) (10.3 and 6.3%, respectively) were not surprising since these two amino acids are often highly conserved in protein families due to their essential role in preserving the protein tertiary structure.

**Fig. 4:**
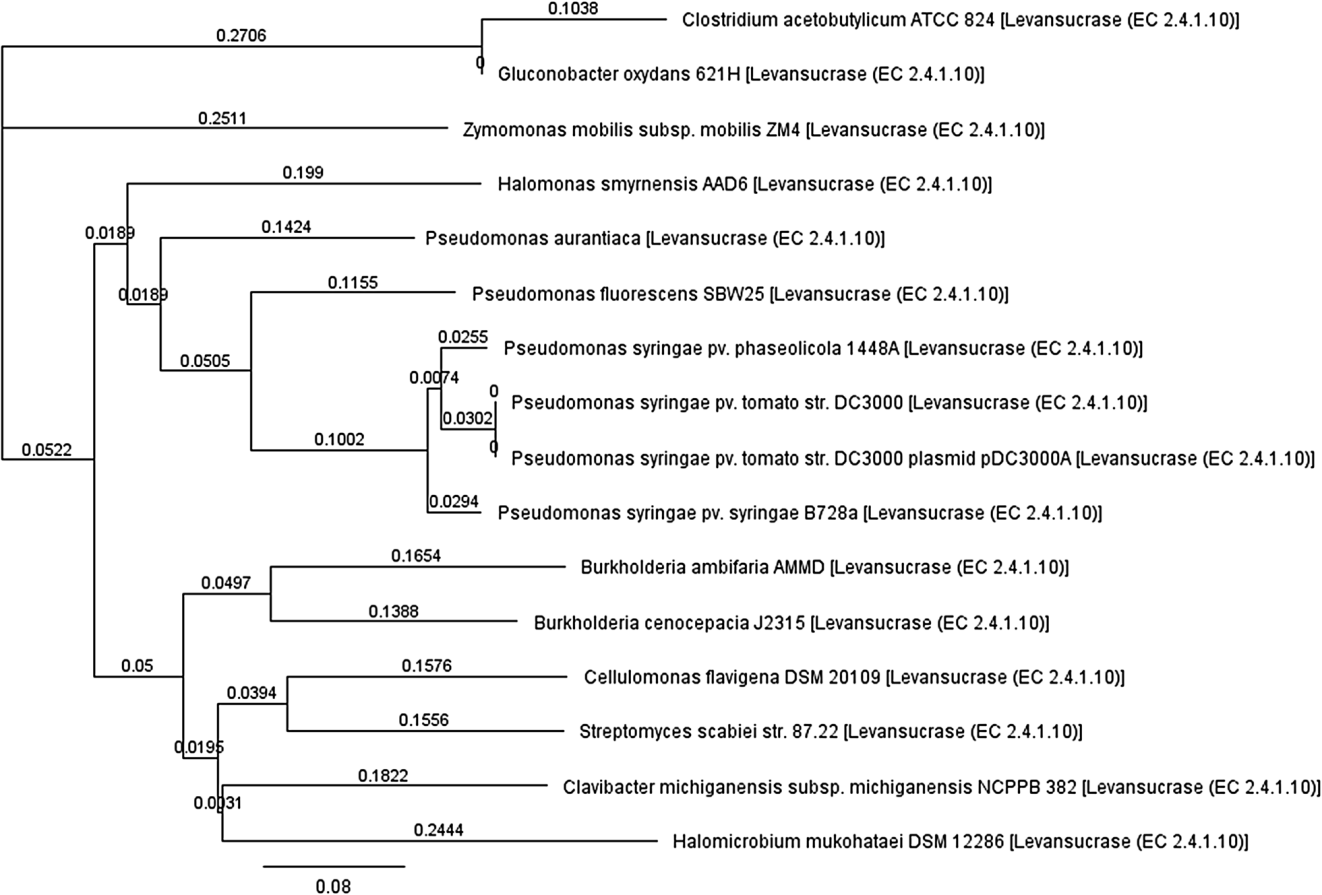
Phylogenetic classification of levansucrase enzymes. The phylogenetic tree was constructed using maximum likelihood method and nucleotide sequences of enzymes taken from NCBI database.

The phylogenetic classification based on nucleotide sequence for a set of bacterial levansucrase enzymes indicated that the most closely related levansucrases to *Hs_SacB* were those from *Pseudomonas* strains (Fig. 4). Similar results were obtained in classification based on the amino acid sequences, where *Hs_SacB* enzyme exhibited high similarities with levansucrases from *Pseudomonas* strains with 96–99% coverage.

Levansucrases follow different secretion routes, and are generally secreted by a signal-peptide-independent pathway in the gram-negative bacteria (Arrieta et al. 2004). Multiple sequence alignment analysis indicated the absence of such a signal-peptide, and pointed out a signal-peptide-independent secretion pathway in *H*. *smyrnensis* AAD6T like other gram-negative bacteria.

### Biosynthesis of Pel exopolysaccharide

One strategy for bacterial adaptation to environmental stresses is to self-encapsulate with a matrix material, primarily composed of secreted extracellular polysaccharides (Sutherland 1998). *H*. *smyrnensis* AAD6T is known to produce high levels of levan EPS (Poli et al. 2009), but there is no experimental study reporting biosynthesis of any other EPSs. The genome analysis revealed a putative Pel polysaccharide gene cluster (PelA, PelB, PelC, PelD, PelE, PelF, and PelG) in *H*. *smyrnensis* AAD6T (Additional file 4: Figure S4). PelB gene, annotated as a conserved hypothetical protein via RAST server, was presumed to encode a polysaccharide biosynthesis protein due to both its position in the genome and amino acid sequence similarity with “biofilm formation protein PelB” of *Pseudomonas mendocina* (99% coverage) and *Pseudomonas aeruginosa* (96% coverage).

Pel polysaccharide is a glucose-rich, cellulose-like polymer, which is essential for the formation of a pellicle at the air–liquid interface. *P*. *aeruginosa* is the most-well known Pel polysaccharide producer, and this bacterium also produces Alginate and Psl polysaccharides as well as Pel polysaccharide, all of which has been shown to be important for biofilm formation (Colvin et al. 2012). In AAD6T genome, Psl related genes were not detected, however, “Alginate lyase precursor (EC 4.2.2.3)” and “Alginate biosynthesis protein Alg8” genes were predicted.

### Biosynthesis of polyhydroxyalkanoates (PHAs)

In addition to genes related to EPS biosynthesis, genes that are involved in intracellular polyhydroxyalkanoate (PHA) biosynthesis were also detected. Fifteen genes related to “Acetyl-CoA fermentation to Butyrate-Polyhydroxybutyrate” metabolism along with “Polyhydroxyalkanoate synthesis repressor PhaR” and “3-ketoacyl-CoA reductase PhaB” genes were present in the draft genome. PHAs are natural polyesters produced as carbon and energy reserve materials by many microorganisms under stressful growth conditions in the presence of excess carbon sources. The stored PHA can be degraded by intracellular depolymerases and metabolized as carbon and energy source as soon as the supply of the limiting nutrient is restored (Philip et al. 2007).

In addition to *H*. *smyrnensis* AAD6T, ten *Halomonas* strains (*H*. *eurihalina*, *H*. *sinaiensis*, *H*. *almeriensis*, *H*. *salina*, *H*. *maura*, *H*. *ventosae*, *H*. *anticariensis*, *H*. *alkaliphila, H*. *almeriensis, H*. *rifensis*) were also reported to secrete EPSs along with PHAs (Amjres et al. 2011; Llamas et al. 2012; Rodriguez-Valera and Lillo 1992). This potential of the bacteria should be considered in the design of bioreactor experiments, since the co-production of EPS and PHA may cause difficulties in bioreactors as they tend to increase the viscosity of the medium rapidly and may also interfere with purification of the polyester (Rodriguez-Valera and Lillo 1992).

### Other features of *H*. *smyrnensis* AAD6T genome

The genome annotation results pointed out several genes and gene clusters associated with other biological processes such as cell division, transcription process, lipopolysaccharide (LPS) biosynthesis, quorum sensing and arsenic resistance (Additional file 7). Briefly, fifteen genes were identified, which encode proteins associated with cell division. A significant portion of these proteins belonged to the Fts (filamentation thermosensitive) family. *H*. *smyrnensis* AAD6T genome presents a Min system, consisting of three proteins MinCDE, which controls the division site selection. The rod-shape of the microorganism is provided by functioning of four rod-shape determining proteins: RodA, MreB, MreC and MreD, which in turn serves as a genetic support to the observations. The genome annotation identified several transcription process associated genes, which show high similarities to the corresponding orthologous of *C*. *salexigens*. 16S, 23S, 5S rRNA and 61 tRNA regions (for all amino acids) were also annotated.

As a gram-negative bacterium, *H*. *smyrnensis* AAD6T possesses LPSs in the external leaflet of its outer membrane. Several genes associated with lipopolysaccharide assembly were identified in *H*. *smyrnensis* AAD6T. Several genes such as LuxS, LuxP, RpoS, RpoN that play roles in cell-density-dependent gene-expression mechanism (quorum sensing) and bacterial cell-to-cell communication were identified.

The *H*. *smyrnensis* AAD6T genome also carries multiple genes that are potentially involved in arsenic resistance, as has also been reported for other *Halomonas* species (Lin et al. 2012; Wolfe-Simon et al. 2011). Presence of these genes suggests that *H*. *smyrnensis* AAD6T is an arsenite-specific expulsion prokaryote rather than a dissimilatory arsenic-reducing prokaryote. The predicted arsenic resistance genes in the genome, as well as scientific reports for arsenic resistance of several *Halomonas* species support the genomic potential of arsenic resistance of *H*. *smyrnensis* AAD6T and put it as a candidate organism for bioremediation studies.

## Conclusions

The microbial genome data form the basis of systems biology research including functional genomics and metabolic engineering studies. Therefore, whole genome analysis of *H*. *smyrnensis* AAD6T was performed. The genome analysis reveals the biotechnological and industrial potential of this levan producing halophilic extremophile. The bacterium was found to have many potential applications in biotechnology not only being a levan producer, but also because of its capacity to produce Pel exopolysaccharide, PHAs, and osmoprotectants as well as its resistance to arsenic.

The genomic information of *H*. *smyrnensis* AAD6T together with the reconstructed genome-scale metabolic model presented here will not only provide additional information to enhance our understanding of the genetic and metabolic network of halophilic bacteria, but also accelerate research on medium design and development of metabolic engineering strategies for enhanced levan biosynthesis, systematical design of strain development strategies for biotechnology applications, and purification and characterization of levansucrase enzyme. Consequently, the information presented here will be a crucial pathfinder towards future in silico and in vivo studies of this organism.

### Nucleotide sequence accession numbers

The genome project for *H*. *smyrnensis* AAD6T has been deposited at DDBJ/EMBL/GenBank under the accession AJKS00000000. This is the second version, with accession numbers AJKS02000001 to AJKS02000034. The 16S rRNA gene and levansucrase (Hs_SacB) gene sequences are available in DDBJ/EMBL/GenBank under the accession numbers DQ131909.2 and KC480580.1, respectively.

## Additional files

Additional file 1:
**Table S1.** The coding sequences, their genomic positions, and function annotation results. Additional file 2:
**Table S2.** Nucleotide and amino acid sequences of predicted coding sequences. Additional file 3:
**Table S3.** Subsystem annotation results of the predicted coding sequences.
**Additional file 4:** Text document including the Figures S1–S5. Additional file 5:
**Table S4.** The distribution of the proteins and associated genes of the global metabolism.
**Additional file 6:** The genome scale metabolic model (*i*KYA1142) of *H*. *smyrnensis* AAD6T.
**Additional file 7:** Text document including the additional information on other cellular processes represented in *H*. *smyrnensis* AAD6T genome.
