# Supplementary material for: Genomic analysis reveals the biotechnological and industrial potential of levan producing halophilic extremophile, Halomonas smyrnensis AAD6T
Source: Springerplus. 2015 Aug 4;4:393. doi: 10.1186/s40064-015-1184-3 (PMC4523562; doi:10.1186/s40064-015-1184-3)
Supplement: Supplementary file 4 — Additional file 4: Text document including the Figures S1–S5. [file 40064_2015_1184_MOESM4_ESM.docx]

**SpringerPlus**

**Genomic analysis reveals the biotechnological and industrial potential of levan producing halophilic extremophile, *Halomonas smyrnensis* AAD6T**

**Elif Sogutcu^1^, Tugba Ozer^1^, Muzaffer Arikan^2^, Zeliha Emrence^2^, Ebru Toksoy Oner^1^, Duran Ustek^3^, Kazim Yalcin Arga^1,§^**

^1^ Department of Bioengineering, Marmara University, Goztepe 34722, Istanbul, Turkey

^2^ Department of Genetics, Institute for Experimental Medicine, Istanbul University, Capa 34093, Istanbul, Turkey

^3^ Department of Medical Genetics, School of Medicine, REMER, Medipol University, 34810, Istanbul, Turkey

^§^Corresponding author:

Assoc. Prof. Dr. Kazim Yalcin Arga

E-mail: kazim.arga@marmara.edu.tr

**Figure S1:** Phylogenetic tree **based on 16S rRNA sequences** for a) the **family *Halomonadaceae*, b) a set of bacterial strains whose whole genome sequences are available.**

**
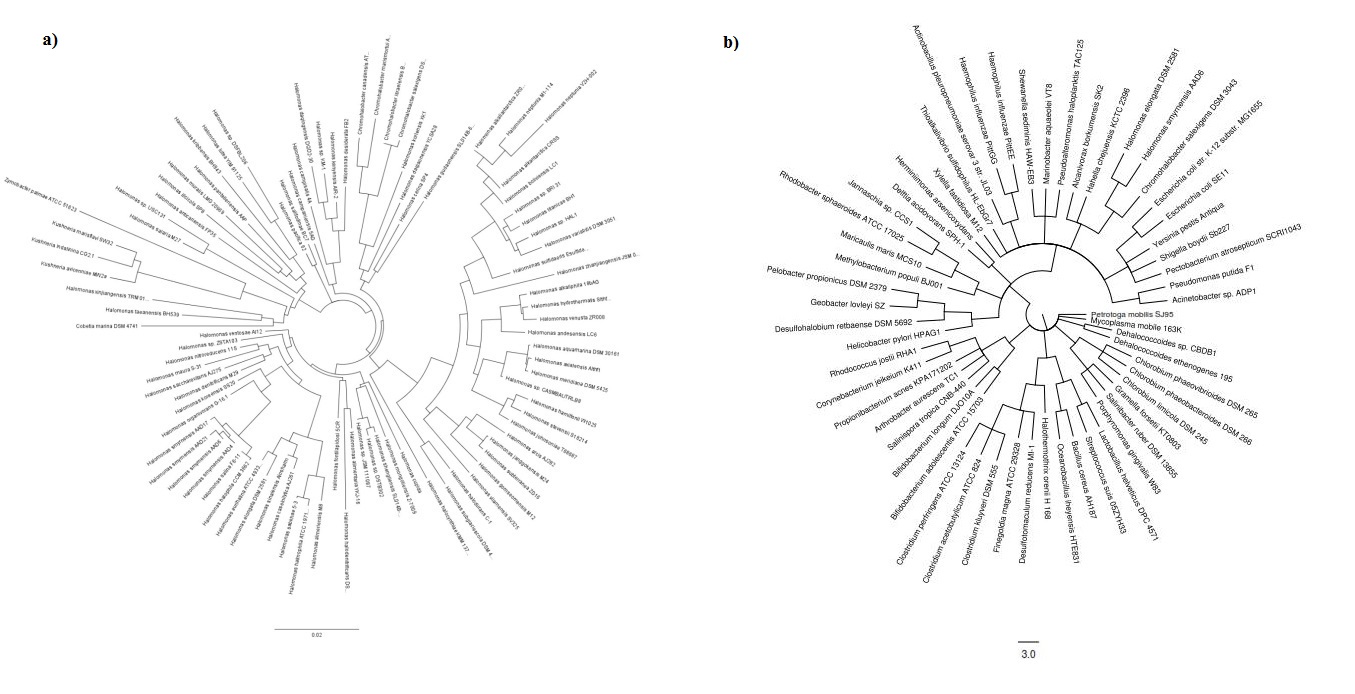
**

**Figure S2**: The proposed Glycine Betaine/Proline Betaine and Choline uptake system in *H. smyrnensis* AAD6T.

**
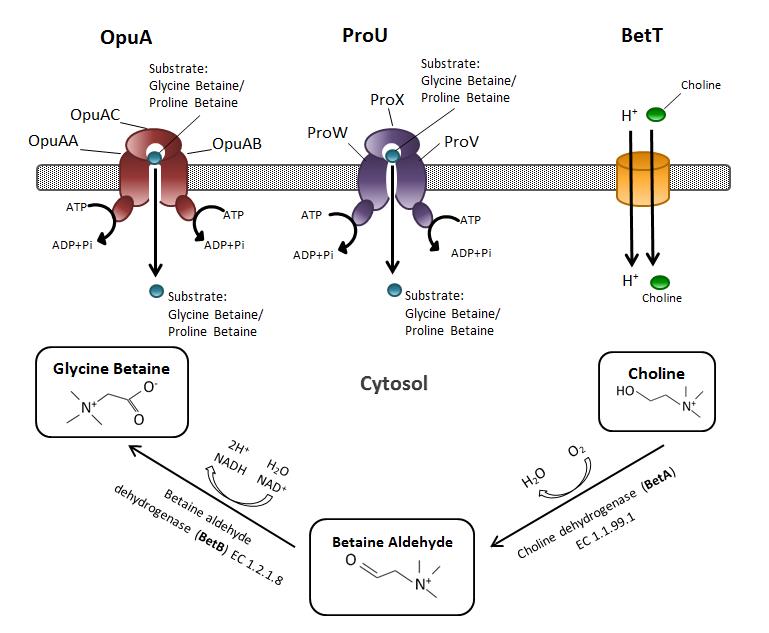
**

**Figure S3**: The amino acid distribution of Hs_SacB enzyme in *H. smyrnensis* AAD6T.

**
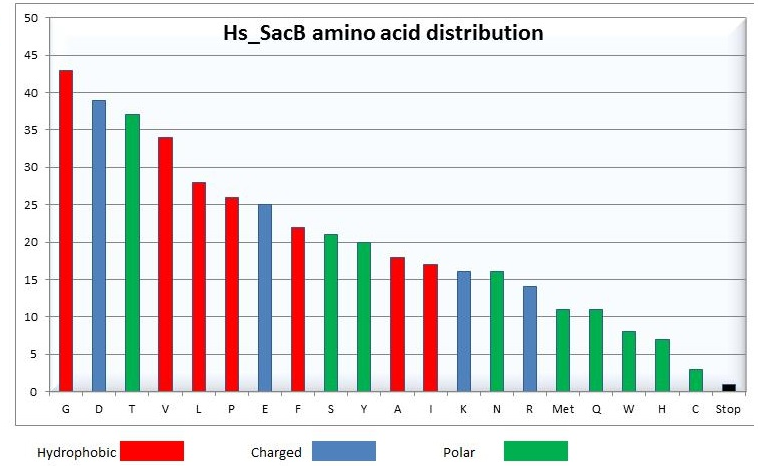
**

**Figure S4**: The gene cluster for Pel polysaccharide biosynthesis in *H. smyrnensis* AAD6T

**
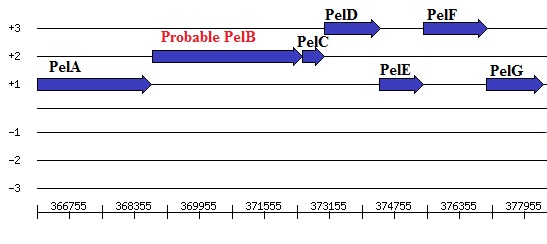
**

**Figure S5:** The proposed model for cell division proteins of *H. smyrnensis* AAD6T. PG: Peptidoglycan, CM: Cellular Membrane

**
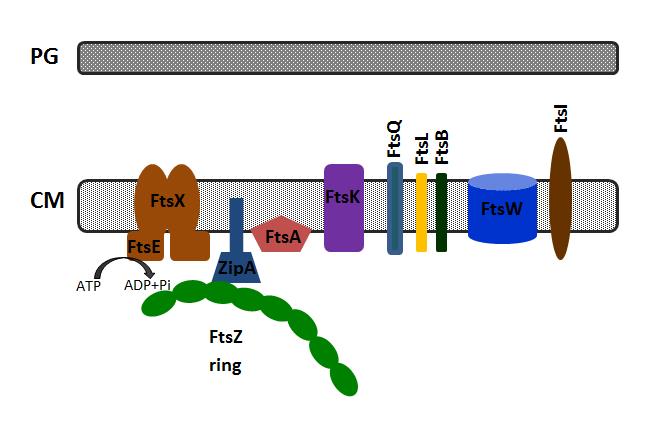
**
