# Supplementary material for: Genomic analysis reveals the biotechnological and industrial potential of levan producing halophilic extremophile, Halomonas smyrnensis AAD6T
Source: Springerplus. 2015 Aug 4;4:393. doi: 10.1186/s40064-015-1184-3 (PMC4523562; doi:10.1186/s40064-015-1184-3)
Supplement: Additional file 5: — Table S4. The distribution of the proteins and associated genes of the global metabolism. [file 40064_2015_1184_MOESM5_ESM.docx]

**SpringerPlus**

**Genomic analysis reveals the biotechnological and industrial potential of levan producing halophilic extremophile, *Halomonas smyrnensis* AAD6T**

**Elif Sogutcu^1^, Tugba Ozer^1^, Muzaffer Arikan^2^, Zeliha Emrence^2^, Ebru Toksoy Oner^1^, Duran Ustek^3^, Kazim Yalcin Arga^1,§^**

^1^ Department of Bioengineering, Marmara University, Goztepe 34722, Istanbul, Turkey

^2^ Department of Genetics, Institute for Experimental Medicine, Istanbul University, Capa 34093, Istanbul, Turkey

^3^ Department of Medical Genetics, School of Medicine, REMER, Medipol University, 34810, Istanbul, Turkey

^§^Corresponding author:

Assoc. Prof. Dr. Kazim Yalcin Arga

E-mail: kazim.arga@marmara.edu.tr

**Table S4: The distribution of the proteins and associated genes of the global metabolism**

| Gene/Enzyme | E.C. Number | Gene Identification |
| --- | --- | --- |
| **Central Carbohydrate Metabolism** | | |
| **Glycolysis and Gluconeogenesis** | | |
| 6-phosphofructokinase | 2.7.1.11 | peg.1123 |
| Triosephosphate isomerase | 5.3.1.1 | peg.163 |
| Fructose-bisphosphate aldolase class II | 4.1.2.13 | peg.2647 |
| 2,3bisphosphoglycerate-independent phosphoglycerate mutase | 5.4.2.1 | peg.313 |
| Glucose-6-phosphate isomerase | 5.3.1.9 | peg.1764  peg.2461 |
| Fructose-bisphosphate aldolase class I | 4.1.2.13 | peg.2654 |
| NADPH-dependent glyceraldehyde-3-phosphate dehydrogenase | 1.2.1.13 | peg.1159 |
| Glucokinase | 2.7.1.2 | peg.2587 |
| Pyruvate kinase | 2.7.1.40 | peg.1553 peg.1840 peg.2459 |
| Phosphoenolpyruvate synthase | 2.7.9.2 | peg.1292 |
| Phosphoglycerate kinase | 2.7.2.3 | peg.2648 |
| Enolase | 4.2.1.11 | peg.763 peg.1841 |
| NAD-dependent glyceraldehyde-3-phosphate dehydrogenase | 1.2.1.12 | peg.1726 peg.2458 |
| **TCA Cycle** | | |
| **Gene/Enzyme** | **E.C. Number** | **Gene Identification** |
| Isocitrate dehydrogenase [NADP] | 1.1.1.42 | peg.1982  peg.2401 |
| Malate dehydrogenase | 1.1.1.37 | peg.1792 peg.1908 |
| Dihydrolipoamide dehydrogenase of 2-oxoglutarate dehydrogenase | 1.8.1.4 | peg.2909 |
| Succinyl-CoA ligase [ADP-forming] alpha chain | 6.2.1.5 | peg.2907 |
| Succinate dehydrogenase flavoprotein subunit | 1.3.99.1 | peg.2913 |
| Aconitate hydratase | 4.2.1.3 | peg.244 peg.245 peg.691 peg.3152 |
| Succinate dehydrogenase iron-sulfur protein | 1.3.99.1 | peg.2912 |
| Dihydrolipoamide succinyltransferase component (E2) of 2-oxoglutarate dehydrogenase complex | 2.3.1.61 | peg.2910 |
| Aconitate hydratase 2 | 4.2.1.3 | peg.1297 |
| Citrate synthase (si) | 2.3.3.1 | peg.209 peg.2916 |
| Fumarate hydratase class I, aerobic | 4.2.1.2 | peg.2728 |
| 2-oxoglutarate dehydrogenase E1 component | 1.2.4.2 | peg.2911 |
| Succinyl-CoA ligase [ADP-forming] beta chain | 6.2.1.5 | peg.2908 |
| Fumarate hydratase class II | 4.2.1.2 | peg.1259 |
| **Pentose phosphate pathway** | | |
| **Gene/Enzyme** | **E.C. Number** | **Gene Identification** |
| Transketolase | 2.2.1.1 | peg.2656 |
| Ribose-phosphate pyrophosphokinase | 2.7.6.1 | peg.654 |
| Ribose 5-phosphate isomerase A | 5.3.1.6 | peg.324 |
| Ribulose-phosphate 3-epimerase | 5.1.3.1 | peg.1070 |
| Glucose-6-phosphate 1-dehydrogenase | 1.1.1.49 | peg.1638 |
| 6-phosphogluconate dehydrogenase, decarboxylating | 1.1.1.44 | peg.2220 |
| 6-phosphogluconolactonase | 3.1.1.31 | peg.1639 |
| Transaldolase | 2.2.1.2 | peg.1720 |
| **Glyoxylate bypass** | | |
| **Gene/Enzyme** | **E.C. Number** | **Gene Identification** |
| Malate synthase G | 2.3.3.9 | peg.2502 |
| Malate dehydrogenase | 1.1.1.37 | peg.1792 peg.1908 |
| Citrate synthase (si) | 2.3.3.1 | peg.209 peg.2916 |
| Aconitate hydratase 2 | 4.2.1.3 | peg.1297 |
| Isocitrate lyase | 4.1.3.1 | peg.2391 |
| Aconitate hydratase | 4.2.1.3 | peg.244 peg.245 peg.691  peg.3152 |
| **Glycolate, glyoxylate interconversions** | | |
| **Gene/Enzyme** | **E.C. Number** | **Gene Identification** |
| Glycolate dehydrogenase, iron-sulfur subunit GlcF | 1.1.99.14 | peg.2290 |
| Hydroxypyruvate reductase | 1.1.1.81 | peg.2637 |
| Glyoxylate reductase | 1.1.1.79 | peg.2637 |
| Glyoxylate reductase | 1.1.1.26 | peg.2637 |
| Phosphoglycolate phosphatase | 3.1.3.18 | peg.1074 |
| Glycolate dehydrogenase, subunit GlcD | 1.1.99.14 | peg.2288 |
| Glycolate dehydrogenase, FAD-binding subunit GlcE | 1.1.99.14 | peg.2289 |
| Hypothetical protein GlcG in glycolate utilization operon |  | peg.2291 |
| **Pyruvate metabolism** | | |
| **Gene/Enzyme** | **E.C. Number** | **Gene Identification** |
| Oxaloacetate decarboxylase gamma chain | 4.1.1.3 | peg.774 |
| Oxaloacetate decarboxylase alpha chain | 4.1.1.3 | peg.775 |
| D-malic enzyme | 1.1.1.83 | peg.1845  peg.2311 |
| Oxaloacetate decarboxylase beta chain | 4.1.1.3 | peg.776 |
| Phosphoenolpyruvate carboxykinase [ATP] | 4.1.1.49 | peg.1798 |
| Two-component response regulator, malate | 2.7.3.- | peg.794 |
| Two-component sensor histidine kinase, malate | 2.7.3.- | peg.793 |
| Malate Na(+) symporter |  | peg.792 |
| NAD-dependent malic enzyme | 1.1.1.38 | peg.3153 |
| Phosphoenolpyruvate carboxylase | 4.1.1.31 | peg.474 |
| NADP-dependent malic enzyme | 1.1.1.40 | peg.748 |
| Pyruvate kinase | 2.7.1.40 | peg.1553 peg.1840 peg.2459 |
| Phosphoenolpyruvate synthase | 2.7.9.2 | peg.1292 |
| Pyruvate dehydrogenase E1 component | 1.2.4.1 | peg.889 |
| Acetate permease ActP (cation/acetate symporter) |  | peg.906 |
| Acetyl-coenzyme A  synthetase | 6.2.1.1 | peg.898 peg.2824 |
| Protein acetyltransferase |  | peg.2800 |
| NAD-dependent protein deacetylase of SIR2 family |  | peg.2187 peg.2630 |
| Acylphosphate phosphohydrolase, putative | 3.6.1.7 | peg.2734 |
| Aldehyde dehydrogenase | 1.2.1.3 | peg.411 peg.1181 peg.1388 peg.1516 peg.1579 peg.1890 peg.1895 peg.2063 |
| Dihydrolipoamide acetyltransferase component of pyruvate dehydrogenase complex | 2.3.1.12 | peg.890 |
| **Methylglyoxal Metabolism** | | |
| **Gene/Enzyme** | **E.C. Number** | **Gene Identification** |
| Methylglyoxal synthase | 4.2.3.3 | peg.2460 |
| Lactoylglutathione lyase | 4.4.1.5 | peg.223 peg.2210 |
| Aldehyde dehydrogenase B | 1.2.1.22 | peg.256 |
| Aldehyde dehydrogenase | 1.2.1.3 | peg.411 peg.1181 peg.1388 peg.1516 peg.1579 peg.1890 peg.1895 peg.2063 peg.411 peg.1181 peg.1388 peg.1516 peg.1579 peg.1890 peg.1895 peg.2063 |
| Hydroxyacylglutathione hydrolase | 3.1.2.6 | peg.2832 |
| **Pyruvate Alanine Serine Interconversions** | | |
| **Gene/Enzyme** | **E.C. Number** | **Gene Identification** |
| Branched-chain amino acid aminotransferase | 2.6.1.42 | peg.2197 |
| Omega-amino acid--pyruvate aminotransferase | 2.6.1.18 | peg.39 |
| Alanine dehydrogenase | 1.4.1.1 | peg.3155 |
| L-serine dehydratase | 4.3.1.17 | peg.990 |
| Alanine racemase | 5.1.1.1 | peg.2574 |
| D-amino acid dehydrogenase small subunit | 1.4.99.1 | peg.1211 peg.2883 |
| **Entner-Doudoroff Pathway** | | |
| **Gene/Enzyme** | **E.C. Number** | **Gene Identification** |
| Glucose dehydrogenase PQQ-dependent | 1.1.5.2 | peg.3145 |
| 2 3-bisphosphoglycerate-independent phosphoglycerate mutase | 5.4.2.1 | peg.313 |
| Glucose-6-phosphate 1-dehydrogenase | 1.1.1.49 | peg.1638 |
| 2-dehydro-3-deoxyphosphogluconate aldolase | 4.1.2.14 | peg.1640 |
| Gluconate dehydratase | 4.2.1.39 | peg.1633 |
| 6-phosphogluconolactonase eukaryotic type | 3.1.1.31 | peg.1639 |
| Gluconolactonase | 3.1.1.17 | peg.1636 peg.2314 |
| Gluconokinase | 2.7.1.12 | peg.88 |
| Glucokinase | 2.7.1.2 | peg.2587 |
| Pyruvate kinase | 2.7.1.40 | peg.1553 peg.1840  peg.2459 |
| Phosphoglycerate kinase | 2.7.2.3 | peg.2648 |
| Phosphogluconate dehydratase | 4.2.1.12 | peg.2586 |
| NAD-dependent glyceraldehyde-3-phosphate dehydrogenase | 1.2.1.12 | peg.1726 peg.2458 |
| Enolase | 4.2.1.11 | peg.763 peg.1841 |
| **Dehydrogenase complexes** | | |
| **Gene/Enzyme** | **E.C. Number** | **Gene Identification** |
| Pyruvate dehydrogenase E1 component | 1.2.4.1 | peg.889 |
| Branched-chain alpha-keto acid dehydrogenase E1 component alpha subunit | 1.2.4.4 | peg.2858 |
| Dihydrolipoamide dehydrogenase of 2-oxoglutarate dehydrogenase | 1.8.1.4 | peg.2909 |
| Dihydrolipoamide succinyltransferase component (E2) of 2-oxoglutarate dehydrogenase complex | 2.3.1.61 | peg.2910 |
| Dihydrolipoamide acyltransferase component of branched-chain alpha-keto acid dehydrogenase complex | 2.3.1.168 | peg.2856 |
| Branched-chain alpha-keto acid dehydrogenase E1 component beta subunit | 1.2.4.4 | peg.2857 |
| Dihydrolipoamide acetyltransferase component of pyruvate dehydrogenase complex | 2.3.1.12 | peg.890 |
| **Serine-glyoxylate cycle** | | |
| **Gene/Enzyme** | **E.C. Number** | **Gene Identification** |
| Malate dehydrogenase | 1.1.1.37 | peg.1792 peg.1908 |
| Serine hydroxymethyltransferase | 2.1.2.1 | peg.1012 peg.2550 |
| Succinyl-CoA:3-ketoacid-coenzyme A transferase subunit B | 2.8.3.5 | peg.2705 |
| Succinate dehydrogenase flavoprotein subunit | 1.3.99.1 | peg.2913 |
| Serine-pyruvate aminotransferase/archaeal aspartate aminotransferase |  | peg.2958 |
| Aconitate hydratase | 4.2.1.3 | peg.244  peg.245  peg.691 peg.3152 |
| Acetoacetyl-CoA reductase | 1.1.1.36 | peg.2987 |
| Succinate dehydrogenase iron-sulfur protein | 1.3.99.1 | peg.2912 |
| Citrate synthase (si) | 2.3.3.1 | peg.209 peg.2916 |
| Succinyl-CoA:3-ketoacid-coenzyme A transferase subunit A | 2.8.3.5 | peg.2704 |
| Methenyltetrahydrofolate cyclohydrolase | 3.5.4.9 | peg.2878 |
| Enolase | 4.2.1.11 | peg.763 peg.1841 |
| Methylcrotonyl-CoA carboxylase carboxyl transferase subunit | 6.4.1.4 | peg.1453 |
| 5-formyltetrahydrofolate cyclo-ligase | 6.3.3.2 | peg.322 |
| Phosphoenolpyruvate carboxykinase [ATP] | 4.1.1.49 | peg.1798 |
| Methylcrotonyl-CoA carboxylase biotin-containing subunit | 6.4.1.4 | peg.1455 |
| Methylenetetrahydrofolate dehydrogenase (NADP+) | 1.5.1.5 | peg.2878 |
| cytosolic long-chain acyl-CoA thioester hydrolase family protein |  | peg.403 peg.2263 |
| Acetyl-CoA acetyltransferase | 2.3.1.9 | peg.114  peg.153 peg.1731 |
| Succinyl-CoA ligase [ADP-forming] alpha chain | 6.2.1.5 | peg.2907 |
| 3-ketoacyl-CoA thiolase | 2.3.1.16 | peg.114  peg.153 |
| Isocitrate lyase | 4.1.3.1 | peg.2391 |
| Fumarate hydratase class I aerobic | 4.2.1.2 | peg.2728 |
| Succinyl-CoA ligase [ADP-forming] beta chain | 6.2.1.5 | peg.2908 |
| Glycerate kinase | 2.7.1.31 | peg.1844 |
| Formyltetrahydrofolate deformylase | 3.5.1.10 | peg.1017 peg.3092 |
| **Di- and oligosaccharides** | | |
| **Maltose and Maltodextrin Utilization** | | |
| **Gene/Enzyme** | **E.C. Number** | **Gene Identification** |
| Putative sucrose phosphorylase | 2.4.1.7 | peg.2954 |
| Maltodextrin glucosidase | 3.2.1.20 | peg.1587 peg.1589 |
| Maltose/maltodextrin ABC transporter permease protein MalF |  | peg.1591 |
| Maltose/maltodextrin ABC transporter substrate binding periplasmic protein MalE |  | peg.1592 |
| Maltose/maltodextrin transport ATP-binding protein MalK | 3.6.3.19 | peg.1588 |
| Maltose/maltodextrin ABC transporter permease protein MalG |  | peg.1590 |
| Maltoporin (maltose/maltodextrin high-affinity receptor phage lambda receptor protein) |  | peg.1274  peg.1593 |
| **Methylcitrate cycle** | | |
| **Gene/Enzyme** | **E.C. Number** | **Gene Identification** |
| Methylisocitrate lyase | 4.1.3.30 | peg.1094 |
| 2-methylisocitrate dehydratase | 4.2.1.99 | peg.1297 |
| Propionate catabolism operon transcriptional regulator of GntR family [predicted] |  | peg.1093 |
| 2-methylcitrate synthase | 2.3.3.5 | peg.1095 |
| 2-methylcitrate dehydratase FeS dependent | 4.2.1.79 | peg.1096 |
| **Propionate-CoA to Succinate Module** | | |
| **Gene/Enzyme** | **E.C. Number** | **Gene Identification** |
| Methylisocitrate lyase | 4.1.3.30 | peg.1094 |
| Aconitate hydratase 2 | 4.2.1.3 | peg.1297 |
| 2-methylisocitrate dehydratase | 4.2.1.99 | peg.1297 |
| Propionate catabolism operon transcriptional regulator of GntR family [predicted] |  | peg.1093 |
| 2-methylcitrate synthase | 2.3.3.5 | peg.1095 |
| 2-methylcitrate dehydratase FeS dependent | 4.2.1.79 | peg.1096 |
| Aconitate hydratase | 4.2.1.3 | peg.244 peg.245 peg.691 peg.3152 |
| **Glycerate metabolism** | | |
| **Gene/Enzyme** | **E.C. Number** | **Gene Identification** |
| Hydroxypyruvate isomerase | 5.3.1.22 | peg.1550 peg.1559 |
| Hydroxypyruvate reductase | 1.1.1.81 | peg.2637 |
| 2-hydroxy-3-oxopropionate reductase | 1.1.1.60 | peg.1552 peg.1839 |
| Glyoxylate carboligase | 4.1.1.47 | peg.1549 |
| Pyruvate kinase | 2.7.1.40 | peg.1553 peg.1840 peg.2459 |
| Tartrate decarboxylase | 4.1.1.73 | peg.1845 peg.2311 |
| Glycerate kinase | 2.7.1.31 | peg.1844 |
| **Butanol Biosynthesis** | | |
| **Gene/Enzyme** | **E.C. Number** | **Gene Identification** |
| Alcohol dehydrogenase | 1.1.1.1 | peg.41  peg.415 peg.842 peg.1424 peg.1436 peg.1517 peg.1527 |
| Enoyl-CoA hydratase | 4.2.1.17 | peg.115 peg.1521 |
| 3-hydroxybutyryl-CoA dehydrogenase | 1.1.1.157 | peg.2942 |
| Acetyl-CoA acetyltransferase | 2.3.1.9 | peg.114 peg.153 peg.1731 |
| **Amino Acids And Derivatives/Alanine Serine And Glycine** | | |
| **Glycine and Serine Utilization** | | |
| **Gene/Enzyme** | **E.C. Number** | **Gene Identification** |
| D-3-phosphoglycerate dehydrogenase | 1.1.1.95 | peg.292 peg.1598 peg.2737 peg.3179 |
| Glycine and Serine Utilization | 3.1.3.3 | peg.1760 peg.1791 peg.2055 |
| Serine hydroxymethyltransferase | 2.1.2.1 | peg.1012 peg.2550 |
| Seryl-tRNA synthetase | 6.1.1.11 | peg.1086 |
| Glycine and Serine Utilization | 2.6.1.52 | peg.3121 |
| D-serine dehydratase transcriptional activator |  | peg.1899 |
| Glycine cleavage system H protein |  | peg.2693 |
| Glycine dehydrogenase [decarboxylating] (glycine cleavage system P protein) | 1.4.4.2 | peg.2692 |
| 2-amino-3-ketobutyrate coenzyme A ligase | 2.3.1.29 | peg.630 |
| L-serine dehydratase | 4.3.1.17 | peg.990 |
| Aminomethyltransferase (glycine cleavage system T protein) | 2.1.2.10 | peg.2696 |
| Glycerate kinase | 2.7.1.31 | peg.1844 |
| **Glycine cleavage system** | | |
| **Gene/Enzyme** | **E.C. Number** | **Gene Identification** |
| Glycine cleavage system H protein |  | peg.2693 |
| Glycine dehydrogenase [decarboxylating] (glycine cleavage system P protein) | 1.4.4.2 | peg.2692 |
| Glycine cleavage system transcriptional activator GcvA |  | peg.427 peg.2305 |
| Sodium/glycine symporter GlyP |  | peg.2695 |
| Aminomethyltransferase (glycine cleavage system T protein) | 2.1.2.10 | peg.2696 |
| **Glycine Biosynthesis** | | |
| **Gene/Enzyme** | **E.C. Number** | **Gene Identification** |
| 2-amino-3-ketobutyrate coenzyme A ligase | 2.3.1.29 | peg.630 |
| Serine hydroxymethyltransferase | 2.1.2.1 | peg.1012 peg.2550 |
| L-threonine 3-dehydrogenase | 1.1.1.103 | peg.631 |
| **Serine Biosynthesis** | | |
| **Gene/Enzyme** | **E.C. Number** | **Gene Identification** |
| D-3-phosphoglycerate dehydrogenase | 1.1.1.95 | peg.292 peg.1598 peg.2737 peg.3179 |
| Phosphoserine phosphatase | 3.1.3.3 | peg.1760 peg.1791 peg.2055 |
| Serine hydroxymethyltransferase | 2.1.2.1 | peg.1012 peg.2550 |
| Phosphoserine aminotransferase | 2.6.1.52 | peg.3121 |
| **Alanine biosynthesis** | | |
| **Gene/Enzyme** | **E.C. Number** | **Gene Identification** |
| Iron binding protein IscA for iron-sulfur cluster assembly |  | peg.2160 |
| HTH-type transcriptional regulator IlvY |  | peg.3199 |
| Branched-chain amino acid aminotransferase | 2.6.1.42 | peg.2197 |
| Cysteine desulfurase | 2.8.1.7 | peg.2159 |
| Cysteine desulfurase SufS subfamily | 2.8.1.7 | peg.76 |
| Alanine racemase | 5.1.1.1 | peg.2574 |
| Iron-sulfur cluster regulator IscR |  | peg.80  peg.2158 |
| **Proline 4-hydroxyproline uptake and utilization** | | |
| **Gene/Enzyme** | **E.C. Number** | **Gene Identification** |
| Proline dehydrogenase (Proline oxidase) | 1.5.99.8 | peg.60  peg.1402 |
| Predicted regulator PutR for proline utilization GntR family |  | peg.113 |
| Ketoglutarate semialdehyde dehydrogenase | 1.2.1.26 | peg.2135 |
| Delta-1-pyrroline-5-carboxylate dehydrogenase | 1.5.1.12 | peg.60  peg.1402 |
| **Proline Synthesis** | | |
| **Gene/Enzyme** | **E.C. Number** | **Gene Identification** |
| Pyrroline-5-carboxylate reductase | 1.5.1.2 | peg.138 peg.1906 |
| Gamma-glutamyl phosphate reductase | 1.2.1.41 | peg.1131 |
| **A Hypothetical Protein Related to Proline Metabolism** | | |
| **Gene/Enzyme** | **E.C. Number** | **Gene Identification** |
| Hypothetical protein YggS proline synthase co-transcribed bacterial homolog PROSC |  | peg.137 |
| Pyrroline-5-carboxylate reductase | 1.5.1.2 | peg.138  peg.1906 |
| **Arginine and Ornithine Degradation** | | |
| **Gene/Enzyme** | **E.C. Number** | **Gene Identification** |
| Ornithine cyclodeaminase | 4.3.1.12 | peg.259 |
| Arginine/ornithine ABC transporter permease protein AotQ |  | peg.2317 |
| Delta-1-pyrroline-5-carboxylate dehydrogenase | 1.5.1.12 | peg.60  peg.1402 |
| Succinylglutamic semialdehyde dehydrogenase | 1.2.1.71 | peg.2103 |
| Agmatine deiminase | 3.5.3.12 | peg.544 |
| Succinylarginine dihydrolase | 3.5.3.23 | peg.2102 |
| Histidine ABC transporter permease protein HisQ | 3.A.1.3.1 | peg.1888 |
| Biosynthetic arginine decarboxylase | 4.1.1.19 | peg.3144 |
| Arginine N-succinyltransferase | 2.3.1.109 | peg.2104 |
| Histidine ABC transporter permease protein HisM | 3.A.1.3.1 | peg.2318 |
| **Arginine Biosynthesis -- gjo** | | |
| **Gene/Enzyme** | **E.C. Number** | **Gene Identification** |
| N-succinyl-L L-diaminopimelate desuccinylase | 3.5.1.18 | peg.698 |
| Acetylornithine deacetylase | 3.5.1.16 | peg.2028 |
| Glutamate N-acetyltransferase | 2.3.1.35 | peg.547 |
| Acetylglutamate kinase | 2.7.2.8 | peg.3165 |
| N-acetylglutamate synthase | 2.3.1.1 | peg.547  peg.810 |
| Arginine pathway regulatory protein ArgR repressor of arg regulon |  | peg.1543 peg.2112 |
| Argininosuccinate lyase | 4.3.2.1 | peg.2470 |
| Argininosuccinate synthase | 6.3.4.5 | peg.1833 |
| **Urea carboxylase and Allophanate hydrolase cluster** | | |
| **Gene/Enzyme** | **E.C. Number** | **Gene Identification** |
| Allophanate hydrolase 2 subunit 1 | 3.5.1.54 | peg.858 peg.2849 |
| Lactam utilization protein LamB |  | peg.859 peg.2850 |
| Urea carboxylase without Allophanate hydrolase 2 domains | 6.3.4.6 | peg.857 |
| Allophanate hydrolase 2 subunit 2 | 3.5.1.54 | peg.858 peg.2848 |
| **Arginine Deiminase Pathway** | | |
| **Gene/Enzyme** | **E.C. Number** | **Gene Identification** |
| Arginine pathway regulatory protein ArgR repressor of arg regulon |  | peg.1543 peg.2112 |
| Ornithine carbamoyltransferase | 2.1.3.3 | peg.1836 |
| **Urease subunits** | | |
| **Gene/Enzyme** | **E.C. Number** | **Gene Identification** |
| Urease accessory protein UreD |  | peg.1240 |
| Urease accessory protein UreF |  | peg.1245 |
| Urease beta subunit | 3.5.1.5 | peg.1242 |
| Urease accessory protein UreG |  | peg.1246 |
| Urease gamma subunit | 3.5.1.5 | peg.1241 |
| Urease accessory protein UreE |  | peg.1244 |
| Urease alpha subunit | 3.5.1.5 | peg.1243 |
| **Arginine Biosynthesis extended** | | |
| **Gene/Enzyme** | **E.C. Number** | **Gene Identification** |
| N-succinyl-L L-diaminopimelate desuccinylase | 3.5.1.18 | peg.698 |
| Acetylornithine deacetylase | 3.5.1.16 | peg.2028 |
| Glutamate N-acetyltransferase | 2.3.1.35 | peg.547 |
| Acetylglutamate kinase | 2.7.2.8 | peg.3165 |
| N-acetylglutamate synthase | 2.3.1.1 | peg.547  peg.810 |
| Arginine pathway regulatory protein ArgR repressor of arg regulon |  | peg.1543 peg.2112 |
| Argininosuccinate lyase | 4.3.2.1 | peg.2470 |
| Argininosuccinate synthase | 6.3.4.5 | peg.1833 |
| N-acetyl-gamma-glutamyl-phosphate reductase | 1.2.1.38 | peg.373 |
| Ornithine carbamoyltransferase | 2.1.3.3 | peg.1836 |
| Acetylornithine aminotransferase | 2.6.1.11 | peg.2106 |
| **Urea decomposition** | | |
| **Gene/Enzyme** | **E.C. Number** | **Gene Identification** |
| Urease accessory protein UreF |  | peg.1245 |
| Urea ABC transporter permease protein UrtC |  | peg.1237 |
| Urea ABC transporter ATPase protein UrtE |  | peg.1239 |
| Urea ABC transporter ATPase protein UrtD |  | peg.1238 |
| Urease gamma subunit | 3.5.1.5 | peg.1241 |
| Urease accessory protein UreE |  | peg.1244 |
| Urea ABC transporter permease protein UrtB |  | peg.1236 |
| Urease alpha subunit | 3.5.1.5 | peg.1243 |
| Urease accessory protein UreD |  | peg.1240 |
| Urease beta subunit | 3.5.1.5 | peg.1242 |
| Urease accessory protein UreG |  | peg.1246 |
| HupE-UreJ family metal transporter |  | peg.1247 |
| **Methionine Biosynthesis** | | |
| **Gene/Enzyme** | **E.C. Number** | **Gene Identification** |
| Methionine ABC transporter ATP-binding protein |  | peg.1281 |
| S-adenosylmethionine synthetase | 2.5.1.6 | peg.2657 |
| O-succinylhomoserine sulfhydrylase | 2.5.1.48 | peg.1204 peg.2844 |
| Serine acetyltransferase | 2.3.1.30 | peg.2157 |
| Homoserine kinase | 2.7.1.39 | peg.3143 |
| Betaine--homocysteine S-methyltransferase | 2.1.1.5 | peg.2348 |
| 5-methyltetrahydrofolate--homocysteine methyltransferase | 2.1.1.13 | peg.2786 |
| Cysteine synthase | 2.5.1.47 | peg.1647 |
| O-acetylhomoserine sulfhydrylase | 2.5.1.49 | peg.1204 peg.1687 peg.2844 |
| S-ribosylhomocysteine lyase | 4.4.1.21 | peg.240 |
| Homoserine dehydrogenase | 1.1.1.3 | peg.3007 |
| Methionine ABC transporter substrate-binding protein |  | peg.1279 |
| Homoserine O-acetyltransferase | 2.3.1.31 | peg.140 |
| S-adenosylhomocysteine nucleosidase | 3.2.2.9 | peg.2058 peg.2879 |
| Methionine ABC transporter permease protein |  | peg.1280 |
| Transcriptional activator MetR |  | peg.2215 |
| **Methionine Degradation** | | |
| **Gene/Enzyme** | **E.C. Number** | **Gene Identification** |
| S-adenosylhomocysteine nucleosidase | 3.2.2.9 | peg.2058 peg.2879 |
| Methionine ABC transporter substrate-binding protein |  | peg.1279 |
| Methionine ABC transporter ATP-binding protein |  | peg.1281 |
| Methionine ABC transporter permease protein |  | peg.1280 |
| S-ribosylhomocysteine lyase | 4.4.1.21 | peg.240 |
| S-adenosylmethionine synthetase | 2.5.1.6 | peg.2657 |
| Pyruvate dehydrogenase E1 component | 1.2.4.1 | peg.889 |
| **Cysteine Biosynthesis** | | |
| **Gene/Enzyme** | **E.C. Number** | **Gene Identification** |
| Sulfate permease Trk-type |  | peg.1996 |
| Cysteine synthase B | 2.5.1.47 | peg.478 |
| Cys regulon transcriptional activator CysB |  | peg.1090 peg.1187 |
| Serine acetyltransferase | 2.3.1.30 | peg.2157 |
| Cysteine synthase | 2.5.1.47 | peg.1647 |
| Phosphoadenylyl-sulfate reductase [thioredoxin] | 1.8.4.8 | peg.1091 |
| Sulfate adenylyltransferase subunit 1 | 2.7.7.4 | peg.1767 |
| Sulfate adenylyltransferase subunit 2 | 2.7.7.4 | peg.1768 |
| Sulfate permease |  | peg.303 peg.1622 peg.2554 |
| Sulfite reductase [NADPH] hemoprotein beta-component | 1.8.1.2 | peg.2789 |
| Sulfate transporter CysZ-type |  | peg.2541 |
| **Lysine Biosynthesis DAP Pathway GJO scratch** | | |
| **Gene/Enzyme** | **E.C. Number** | **Gene Identification** |
| N-succinyl-L L-diaminopimelate desuccinylase | 3.5.1.18 | peg.698 |
| N-acetyl-L L-diaminopimelate deacetylase | 3.5.1.47 | peg.1891 |
| 2 3 4 5-tetrahydropyridine-2 6-dicarboxylate N-succinyltransferase | 2.3.1.117 | peg.699 |
| Dihydrodipicolinate reductase | 1.3.1.26 | peg.172 |
| Diaminopimelate epimerase | 5.1.1.7 | peg.2473 |
| Aspartate-semialdehyde dehydrogenase | 1.2.1.11 | peg.1191 |
| Dihydrodipicolinate synthase | 4.2.1.52 | peg.881 peg.1390 |
| Diaminopimelate decarboxylase | 4.1.1.20 | peg.2472 |
| N-succinyl-L L-diaminopimelate aminotransferase alternative | 2.6.1.17 | peg.701 |
| N-succinyl-L L-diaminopimelate aminotransferase | 2.6.1.17 | peg.2106 |
| Aspartokinase | 2.7.2.4 | peg.771 |
| **Threonine degradation** | | |
| **Gene/Enzyme** | **E.C. Number** | **Gene Identification** |
| Threonine dehydrogenase and related Zn-dependent dehydrogenases |  | peg.1004 peg.1391 |
| L-threonine 3-dehydrogenase | 1.1.1.103 | peg.631 |
| **Threonine and Homoserine Biosynthesis** | | |
| **Gene/Enzyme** | **E.C. Number** | **Gene Identification** |
| Homoserine dehydrogenase | 1.1.1.3 | peg.3007 |
| Aspartate-semialdehyde dehydrogenase | 1.2.1.11 | peg.1191 |
| Aspartate aminotransferase | 2.6.1.1 | peg.143 peg.846 peg.1213 peg.1480 peg.1599 peg.1885 peg.2067 peg.2423 |
| Threonine synthase | 4.2.3.1 | peg.3008 |
| Homoserine kinase | 2.7.1.39 | peg.3143 |
| Aspartokinase | 2.7.2.4 | peg.771 |
| **Lysine Biosynthesis DAP Pathway** | | |
| **Gene/Enzyme** | **E.C. Number** | **Gene Identification** |
| N-succinyl-L L-diaminopimelate desuccinylase | 3.5.1.18 | peg.698 |
| N-acetyl-L L-diaminopimelate deacetylase | 3.5.1.47 | peg.1891 |
| 2 3 4 5-tetrahydropyridine-2 6-dicarboxylate N-succinyltransferase | 2.3.1.117 | peg.699 |
| Dihydrodipicolinate reductase | 1.3.1.26 | peg.172 |
| Diaminopimelate epimerase | 5.1.1.7 | peg.2473 |
| Aspartate-semialdehyde dehydrogenase | 1.2.1.11 | peg.1191 |
| Dihydrodipicolinate synthase | 4.2.1.52 | peg.881 peg.1390 |
| Diaminopimelate decarboxylase | 4.1.1.20 | peg.2472 |
| N-succinyl-L L-diaminopimelate aminotransferase alternative | 2.6.1.17 | peg.701 |
| N-succinyl-L L-diaminopimelate aminotransferase | 2.6.1.17 | peg.2106 |
| Aspartokinase | 2.7.2.4 | peg.771 |
| **Lysine degradation** | | |
| **Gene/Enzyme** | **E.C. Number** | **Gene Identification** |
| Lysine decarboxylase | 4.1.1.18 | peg.1606 |
| L-pipecolate oxidase | 1.5.3.7 | peg.260 |
| Lysine 2 3-aminomutase | 5.4.3.2 | peg.1757 |
| **Isoleucine degradation** | | |
| **Gene/Enzyme** | **E.C. Number** | **Gene Identification** |
| Acyl-CoA dehydrogenase short-chain specific | 1.3.99.2 | peg.664 |
| Branched-chain amino acid aminotransferase | 2.6.1.42 | peg.2197 |
| Branched-chain alpha-keto acid dehydrogenase E1 component alpha subunit | 1.2.4.4 | peg.2858 |
| Enoyl-CoA hydratase | 4.2.1.17 | peg.115 peg.1521 |
| Dihydrolipoamide acyltransferase component of branched-chain alpha-keto acid dehydrogenase complex | 2.3.1.168 | peg.2856 |
| 3-ketoacyl-CoA thiolase | 2.3.1.16 | peg.114  peg.153 |
| Branched-chain alpha-keto acid dehydrogenase E1 component beta subunit | 1.2.4.4 | peg.2857 |
| 3-hydroxyacyl-CoA dehydrogenase | 1.1.1.35 | peg.115  peg.1848 peg.1849 |
| Branched-chain acyl-CoA dehydrogenase | 1.3.99.12 | peg.3104 |
| **Leucine Degradation and HMG-CoA Metabolism** | | |
| **Gene/Enzyme** | **E.C. Number** | **Gene Identification** |
| Hydroxymethylglutaryl-CoA lyase | 4.1.3.4 | peg.1456 |
| Branched-chain amino acid aminotransferase | 2.6.1.42 | peg.2197 |
| Methylglutaconyl-CoA hydratase | 4.2.1.18 | peg.1454 |
| Methylcrotonyl-CoA carboxylase carboxyl transferase subunit | 6.4.1.4 | peg.1453 |
| Branched-chain alpha-keto acid dehydrogenase E1 component alpha subunit | 1.2.4.4 | peg.2858 |
| Methylcrotonyl-CoA carboxylase biotin-containing subunit | 6.4.1.4 | peg.1455 |
| Succinyl-CoA:3-ketoacid-coenzyme A transferase subunit B | 2.8.3.5 | peg.2705 |
| Acetoacetyl-CoA synthetase | 6.2.1.16 | peg.2702 peg.3106 |
| Leucine dehydrogenase | 1.4.1.9 | peg.2733 |
| Dihydrolipoamide acyltransferase component of branched-chain alpha-keto acid dehydrogenase complex | 2.3.1.168 | peg.2856 |
| Isovaleryl-CoA dehydrogenase | 1.3.99.10 | peg.1452 |
| Succinyl-CoA:3-ketoacid-coenzyme A transferase subunit A | 2.8.3.5 | peg.2704 |
| Branched-chain alpha-keto acid dehydrogenase E1 component beta subunit | 1.2.4.4 | peg.2857 |
| Branched-chain acyl-CoA dehydrogenase | 1.3.99.12 | peg.3104 |
| **Valine degradation** | | |
| **Gene/Enzyme** | **E.C. Number** | **Gene Identification** |
| Branched-chain amino acid aminotransferase | 2.6.1.42 | peg.2197 |
| 3-hydroxyisobutyrate dehydrogenase | 1.1.1.31 | peg.3102 |
| Methylmalonate-semialdehyde dehydrogenase | 1.2.1.27 | peg.40  peg.102 peg.103 peg.1524 peg.1867 peg.2344 |
| Branched-chain alpha-keto acid dehydrogenase E1 component alpha subunit | 1.2.4.4 | peg.2858 |
| 3-hydroxyisobutyryl-CoA hydrolase | 3.1.2.4 | peg.3103 |
| Enoyl-CoA hydratase | 4.2.1.17 | peg.115  peg.1521 |
| Dihydrolipoamide acyltransferase component of branched-chain alpha-keto acid dehydrogenase complex | 2.3.1.168 | peg.2856 |
| Branched-chain alpha-keto acid dehydrogenase E1 component beta subunit | 1.2.4.4 | peg.2857 |
| 3-hydroxyacyl-CoA dehydrogenase | 1.1.1.35 | peg.115 peg.1848  peg.1849 |
| Branched-chain acyl-CoA dehydrogenase | 1.3.99.12 | peg.3104 |
| **Leucine Biosynthesis** | | |
| **Gene/Enzyme** | **E.C. Number** | **Gene Identification** |
| 3-isopropylmalate dehydratase small subunit | 4.2.1.33 | peg.1189 |
| 3-isopropylmalate dehydratase large subunit | 4.2.1.33 | peg.1188 |
| 2-isopropylmalate synthase | 2.3.3.13 | peg.1758 |
| Branched-chain amino acid aminotransferase | 2.6.1.42 | peg.2197 |
| 3-isopropylmalate dehydrogenase | 1.1.1.85 | peg.1190 |
| **Chorismate: Intermediate for synthesis of Tryptophan PAPA antibiotics PABA 3-hydroxyanthranilate and more.** | | |
| **Gene/Enzyme** | **E.C. Number** | **Gene Identification** |
| Phosphoribosylformimino-5-aminoimidazole carboxamide ribotide isomerase | 5.3.1.16 | peg.3212 |
| Anthranilate synthase amidotransferase component | 4.1.3.27 | peg.1076 |
| Aminodeoxychorismate lyase | 4.1.3.38 | peg.518 |
| Tryptophan synthase alpha chain | 4.2.1.20 | peg.1198 peg.2151 |
| Anthranilate phosphoribosyltransferase | 2.4.2.18 | peg.1077 |
| Tryptophan synthase beta chain | 4.2.1.20 | peg.1196 peg.1197  peg.2152 |
| Indole-3-glycerol phosphate synthase | 4.1.1.48 | peg.1078 |
| Anthranilate synthase aminase component | 4.1.3.27 | peg.1075 |
| Phosphoribosylanthranilate isomerase | 5.3.1.24 | peg.1195 |
| Para-aminobenzoate synthase aminase component | 2.6.1.85 | peg.1092 |
| Isochorismatase | 3.3.2.1 | peg.2971 |
| Para-aminobenzoate synthase amidotransferase component | 2.6.1.85 | peg.1076 |
| **Tryptophan synthesis** | | |
| **Gene/Enzyme** | **E.C. Number** | **Gene Identification** |
| Anthranilate synthase amidotransferase component | 4.1.3.27 | peg.1076 |
| Aminodeoxychorismate lyase | 4.1.3.38 | peg.518 |
| Tryptophan synthase alpha chain | 4.2.1.20 | peg.1198  peg.2151 |
| Para-aminobenzoate synthase aminase component | 2.6.1.85 | peg.1092 |
| Anthranilate phosphoribosyltransferase | 2.4.2.18 | peg.1077 |
| Tryptophan synthase beta chain | 4.2.1.20 | peg.1196 peg.1197 peg.2152 |
| Indole-3-glycerol phosphate synthase | 4.1.1.48 | peg.1078 |
| Para-aminobenzoate synthase amidotransferase component | 2.6.1.85 | peg.1076 |
| Anthranilate synthase aminase component | 4.1.3.27 | peg.1075 |
| Phosphoribosylanthranilate isomerase | 5.3.1.24 | peg.1195 |
| **Creatine and Creatinine Degradation** | | |
| **Gene/Enzyme** | **E.C. Number** | **Gene Identification** |
| Cytosine deaminase | 3.5.4.1 | peg.95 |
| **Branched-Chain Amino Acid Biosynthesis** | | |
| **Gene/Enzyme** | **E.C. Number** | **Gene Identification** |
| 3-isopropylmalate dehydratase small subunit | 4.2.1.33 | peg.1189 |
| 2-isopropylmalate synthase | 2.3.3.13 | peg.1758 |
| Branched-chain amino acid aminotransferase | 2.6.1.42 | peg.2197 |
| 3-isopropylmalate dehydrogenase | 1.1.1.85 | peg.1190 |
| 3-isopropylmalate dehydratase large subunit | 4.2.1.33 | peg.1188 |
| Leucine dehydrogenase | 1.4.1.9 | peg.2733 |
| Ketol-acid reductoisomerase | 1.1.1.86 | peg.3200 |
| Threonine dehydratase biosynthetic | 4.3.1.19 | peg.323 |
| Acetolactate synthase large subunit | 2.2.1.6 | peg.3197 |
| Dihydroxy-acid dehydratase | 4.2.1.9 | peg.1265 peg.1804 peg.2339 |
| Leucine-responsive regulatory protein regulator for leucine (or lrp) regulon and high-affinity branched-chain amino acid transport system |  | peg.1602 |
| **HMG CoA Synthesis** | | |
| **Gene/Enzyme** | **E.C. Number** | **Gene Identification** |
| Hydroxymethylglutaryl-CoA lyase | 4.1.3.4 | peg.1456 |
| Acetoacetyl-CoA synthetase | 6.2.1.16 | peg.2702 peg.3106 |
| Methylglutaconyl-CoA hydratase | 4.2.1.18 | peg.1454 |
| Methylcrotonyl-CoA carboxylase carboxyl transferase subunit | 6.4.1.4 | peg.1453 |
| Isovaleryl-CoA dehydrogenase | 1.3.99.10 | peg.1452 |
| Methylcrotonyl-CoA carboxylase biotin-containing subunit | 6.4.1.4 | peg.1455 |
| **Common Pathway For Synthesis of Aromatic Compounds (DAHP synthase to chorismate)** | | |
| **Gene/Enzyme** | **E.C. Number** | **Gene Identification** |
| Shikimate kinase I | 2.7.1.71 | peg.756 |
| 5-Enolpyruvylshikimate-3-phosphate synthase | 2.5.1.19 | peg.3119 |
| Shikimate 5-dehydrogenase I alpha | 1.1.1.25 | peg.2172 |
| 3-dehydroquinate dehydratase II | 4.2.1.10 | peg.2250 |
| 2-keto-3-deoxy-D-arabino-heptulosonate-7-phosphate synthase I alpha | 2.5.1.54 | peg.492  peg.729 |
| 3-dehydroquinate synthase | 4.2.3.4 | peg.757 |
| Chorismate synthase | 4.2.3.5 | peg.1056 |
| **Chorismate Synthesis** | | |
| **Gene/Enzyme** | **E.C. Number** | **Gene Identification** |
| 5-Enolpyruvylshikimate-3-phosphate synthase | 2.5.1.19 | peg.3119 |
| Chorismate mutase I | 5.4.99.5 | peg.3120 |
| 3-dehydroquinate dehydratase II | 4.2.1.10 | peg.2250 |
| 2-keto-3-deoxy-D-arabino-heptulosonate-7-phosphate synthase I alpha | 2.5.1.54 | peg.492  peg.729 |
| Prephenate dehydratase | 4.2.1.51 | peg.3120 |
| 3-dehydroquinate synthase | 4.2.3.4 | peg.757 |
| Chorismate synthase | 4.2.3.5 | peg.1056 |
| Shikimate kinase I | 2.7.1.71 | peg.756 |
| Shikimate 5-dehydrogenase I alpha | 1.1.1.25 | peg.2172 |
| Cyclohexadienyl dehydrogenase | 1.3.1.12  1.3.1.43 | peg.3119 |
| **Phenylalanine and Tyrosine Branches from Chorismate** | | |
| **Gene/Enzyme** | **E.C. Number** | **Gene Identification** |
| Chorismate mutase I | 5.4.99.5 | peg.3120 |
| Prephenate dehydratase | 4.2.1.51 | peg.3120 |
| Cyclohexadienyl dehydrogenase | 1.3.1.12  1.3.1.43 | peg.3119 |
| **Aromatic amino acid degradation** | | |
| **Gene/Enzyme** | **E.C. Number** | **Gene Identification** |
| Tryptophan 2 3-dioxygenase | 1.13.11.11 | peg.2122 |
| 4-hydroxyphenylpyruvate dioxygenase | 1.13.11.27 | peg.1224 |
| Phenylalanine hydroxylase transcriptional activator PhhR |  | peg.1216 |
| Phenylalanine-4-hydroxylase | 1.14.16.1 | peg.1215 |
| **Carbohydrates** | | |
| **Acetyl-CoA fermentation to Butyrate** | | |
| **Gene/Enzyme** | **E.C. Number** | **Gene Identification** |
| 3-hydroxybutyryl-CoA epimerase | 5.1.2.3 | peg.115 |
| 3-hydroxybutyryl-CoA dehydratase | 4.2.1.55 | peg.2941 |
| Electron transfer flavoprotein-ubiquinone oxidoreductase | 1.5.5.1 | peg.509 |
| Electron transfer flavoprotein alpha subunit |  | peg.507 peg.1000 |
| Acetyl-CoA acetyltransferase | 2.3.1.9 | peg.114  peg.153 peg.1731 |
| Acetoacetyl-CoA reductase | 1.1.1.36 | peg.2987 |
| Enoyl-CoA hydratase | 4.2.1.17 | peg.115 peg.1521 |
| Electron transfer flavoprotein beta subunit |  | peg.508 peg.1001 |
| 3-hydroxyacyl-CoA dehydrogenase | 1.1.1.35 | peg.115 peg.1848 peg.1849 |
| 3-hydroxybutyryl-CoA dehydrogenase | 1.1.1.157 | peg.2942 |
| **CO2 uptake carboxysome** | | |
| **Gene/Enzyme** | **E.C. Number** | **Gene Identification** |
| Hypothetical transmembrane protein coupled to NADH-ubiquinone oxidoreductase chain 5 homolog |  | peg.1464 |
| Sensory subunit of low CO2-induced protein complex putative |  | peg.2933 |
| RuBisCO operon transcriptional regulator CbbR |  | peg.1143 |
| **Glycerol and Glycerol-3-phosphate Uptake and Utilization** | | |
| **Gene/Enzyme** | **E.C. Number** | **Gene Identification** |
| Glycerol-3-phosphate ABC transporter permease protein UgpE | 3.A.1.1.3 | peg.674 |
| Glycerol-3-phosphate regulon repressor DeoR family |  | peg.1276 |
| Glycerophosphoryl diester phosphodiesterase periplasmic | 3.1.4.46 | peg.2602 |
| Glycerol-3-phosphate dehydrogenase [NAD(P)+] | 1.1.1.94 | peg.1046 |
| Glycerol-3-phosphate ABC transporter permease protein UgpA | 3.A.1.1.3 | peg.673 |
| Glycerol kinase | 2.7.1.30 | peg.1277 |
| GlpG protein (membrane protein of glp regulon) |  | peg.44 |
| Glycerol-3-phosphate ABC transporter periplasmic glycerol-3-phosphate-binding protein | 3.A.1.1.3 | peg.672 |
| Thiosulfate sulfurtransferase GlpE | 2.8.1.1 | peg.979 |
| Glycerophosphoryl diester phosphodiesterase | 3.1.4.46 | peg.676 peg.1295 |
| Aerobic glycerol-3-phosphate dehydrogenase | 1.1.5.3 | peg.1275 |
| Glycerol-3-phosphate ABC transporter ATP-binding protein UgpC | 3.A.1.1.3 | peg.675 |
| **Inositol catabolism** | | |
| **Gene/Enzyme** | **E.C. Number** | **Gene Identification** |
| Inositol transport system sugar-binding protein |  | peg.1876 |
| Inositol transport system permease protein |  | peg.1873 |
| Inositol transport system ATP-binding protein |  | peg.1874 |
| Predicted transcriptional regulator of the myo-inositol catabolic operon |  | peg.1863 |
| uncharacterized domain |  | peg.1864 |
| Sodium/myo-inositol cotransporter |  | peg.2374 |
| Inosose dehydratase | 4.2.1.44 | peg.1866 |
| Inosose isomerase | 5.3.99.- | peg.1556 peg.1877 |
| Myo-inositol 2-dehydrogenase | 1.1.1.18 | peg.1862 peg.1878 |
| 5-deoxy-glucuronate isomerase | 5.3.1.- | peg.1861 |
| 5-keto-2-deoxygluconokinase | 2.7.1.92 | peg.1864 |
| Epi-inositol hydrolase | 3.7.1.- | peg.1865 |
| **Carbon storage regulator** | | |
| **Gene/Enzyme** | **E.C. Number** | **Gene Identification** |
| Carbon storage regulator |  | peg.772 |
| **Alpha-Amylase locus in Streptocococcus** | | |
| **Gene/Enzyme** | **E.C. Number** | **Gene Identification** |
| Maltose/maltodextrin ABC transporter substrate binding periplasmic protein MalE |  | peg.1592 |
| **D-ribose utilization** | | |
| **Gene/Enzyme** | **E.C. Number** | **Gene Identification** |
| Ribokinase | 2.7.1.15 | peg.2590 peg.2736 |
| Ribose 5-phosphate isomerase A | 5.3.1.6 | peg.324 |
| **D-galactonate catabolism** | | |
| **Gene/Enzyme** | **E.C. Number** | **Gene Identification** |
| 2-dehydro-3-deoxyphosphogalactonate aldolase | 4.1.2.21 | peg.1635 |
| 2-dehydro-3-deoxygalactonokinase | 2.7.1.58 | peg.1634 |
| **2-Ketogluconate Utilization** | | |
| **Gene/Enzyme** | **E.C. Number** | **Gene Identification** |
| Epimerase KguE |  | peg.2639 |
| 2-ketogluconate utilization repressor PtxS |  | peg.2640 |
| 2-ketogluconate kinase | 2.7.1.13 | peg.2638 |
| **Deoxyribose and Deoxynucleoside Catabolism** | | |
| **Gene/Enzyme** | **E.C. Number** | **Gene Identification** |
| Purine nucleoside phosphorylase | 2.4.2.1 | peg.3222 |
| Ribokinase | 2.7.1.15 | peg.2590 peg.2736 |
| Phosphopentomutase | 5.4.2.7 | peg.3221 |
| Nucleoside permease NupC |  | peg.2968 |
| Deoxyribose operon repressor DeoR family |  | peg.2966 |
| Thymidine phosphorylase | 2.4.2.4 | peg.2970 |
| Putative deoxyribonuclease YjjV |  | peg.2722 |
| Deoxyribose-phosphate aldolase | 4.1.2.4 | peg.2969 |
| **D-gluconate and ketogluconates metabolism** | | |
| **Gene/Enzyme** | **E.C. Number** | **Gene Identification** |
| Predicted gluconate TRAP family transporter DctQ subunit |  | peg.90 |
| 2-ketogluconate kinase | 2.7.1.13 | peg.2638 |
| Glucose dehydrogenase PQQ-dependent | 1.1.5.2 | peg.3145 |
| Predicted gluconate TRAP family transporter DctM subunit |  | peg.89 |
| 2-ketoaldonate reductase broad specificity | 1.1.1.215  1.1.1.- | peg.2637 |
| Gluconate dehydratase | 4.2.1.39 | peg.1633 |
| 6-phosphogluconate dehydrogenase decarboxylating | 1.1.1.44 | peg.2220 |
| Gluconokinase | 2.7.1.12 | peg.88 |
| 2 5-diketo-D-gluconic acid reductase B | 1.1.1.274 | peg.430 peg.1400 |
| **Fructose utilization** | | |
| **Gene/Enzyme** | **E.C. Number** | **Gene Identification** |
| Phosphoenolpyruvate-protein phosphotransferase of PTS system | 2.7.3.9 | peg.2601 |
| PTS system fructose-specific IIC component | 2.7.1.69 | peg.1642 |
| Fructose repressor FruR LacI family |  | peg.2600 |
| PTS system fructose-specific IIB component | 2.7.1.69 | peg.1642 |
| Transaldolase | 2.2.1.2 | peg.1720 |
| 1-phosphofructokinase | 2.7.1.56 | peg.1643 |
| PTS system fructose-specific IIA component | 2.7.1.69 | peg.2601 |
| Fructose-specific phosphocarrier protein HPr | 2.7.1.69 | peg.2601 |
| **Cell Division and Cell Cycle** | | |
| **Bacterial Cytoskeleton** | | |
| **Gene/Enzyme** | **E.C. Number** | **Gene Identification** |
| Septum site-determining protein MinC |  | peg.2989 |
| Cell division protein FtsI [Peptidoglycan synthetase] | 2.4.1.129 | peg.563 |
| Cell division protein FtsW |  | peg.558 |
| Chromosome (plasmid) partitioning protein ParB |  | peg.381 |
| Stage 0 sporulation protein J |  | peg.381 |
| Rod shape-determining protein MreD |  | peg.608 |
| Cell division protein FtsL |  | peg.564 |
| Cell division protein FtsK |  | peg.2407 |
| Septum formation protein Maf |  | peg.607 |
| Septum site-determining protein MinD |  | peg.2990 |
| Chromosome (plasmid) partitioning protein ParA |  | peg.380  peg.1658 peg.2043 |
| Rod shape-determining protein RodA |  | peg.1136 |
| Cell division protein FtsZ | 3.4.24.- | peg.552 peg.2513 |
| Cell division protein FtsA |  | peg.553 |
| Rod shape-determining protein MreB |  | peg.610 |
| Rod shape-determining protein MreC |  | peg.609 |
| Cell division protein FtsQ |  | peg.554 |
| Sporulation initiation inhibitor protein Soj |  | peg.380 |
| Cell division topological specificity factor MinE |  | peg.2991 |
| Cell division protein ZipA |  | peg.789 |
| **Cell Wall and Capsule** | | |
| **dTDP-rhamnose synthesis** | | |
| **Gene/Enzyme** | **E.C. Number** | **Gene Identification** |
| dTDP-4-dehydrorhamnose reductase | 1.1.1.133 | peg.3038 |
| dTDP-glucose 4 6-dehydratase | 4.2.1.46 | peg.2889 |
| Glucose-1-phosphate thymidylyltransferase | 2.7.7.24 | peg.971 |
| **Capsular heptose biosynthesis** | | |
| **Gene/Enzyme** | **E.C. Number** | **Gene Identification** |
| Phosphoheptose isomerase | 5.3.1.- | peg.569 |
| D-glycero-D-manno-heptose 1 7-bisphosphate phosphatase | 3.1.1.- | peg.348 |
| **Capsular Polysaccharides Biosynthesis and Assembly** | | |
| **Gene/Enzyme** | **E.C. Number** | **Gene Identification** |
| Polysaccharide export lipoprotein Wza |  | peg.3 |
| Tyrosine-protein kinase Wzc | 2.7.10.2 | peg.1 |
| Oxidoreductase short-chain dehydrogenase/reductase family | 1.1.1.- | peg.3030 |
| Capsular polysaccharide export system protein KpsC |  | peg.3187 |
| Low molecular weight protein-tyrosine-phosphatase Wzb | 3.1.3.48 | peg.2 |
| **Rhamnose containing glycans** | | |
| **Gene/Enzyme** | **E.C. Number** | **Gene Identification** |
| UDP-glucose 4-epimerase | 5.1.3.2 | peg.196  peg.325  peg.1668 peg.2894 |
| dTDP-4-dehydrorhamnose reductase | 1.1.1.133 | peg.3038 |
| dTDP-glucose 4 6-dehydratase | 4.2.1.46 | peg.2889 |
| Glucose-1-phosphate thymidylyltransferase | 2.7.7.24 | peg.971 |
| Capsular polysaccharide export system protein KpsC |  | peg.3187 |
| **Sialic Acid Metabolism** | | |
| **Gene/Enzyme** | **E.C. Number** | **Gene Identification** |
| Glucosamine-1-phosphate N-acetyltransferase | 2.3.1.157 | peg.395 |
| Glucosamine--fructose-6-phosphate aminotransferase [isomerizing] | 2.6.1.16 | peg.396 |
| Phosphoglucosamine mutase | 5.4.2.10 | peg.164 peg.3164 |
| N-acetylglucosamine-1-phosphate uridyltransferase | 2.7.7.23 | peg.395 |
| TRAP-type transport system small permease component predicted N-acetylneuraminate transporter |  | peg.1567  peg.2492 peg.2634 |
| N-acylglucosamine 2-epimerase | 5.1.3.8 | peg.2209 |
| **Lipopolysaccharide assembly** | | |
| **Gene/Enzyme** | **E.C. Number** | **Gene Identification** |
| Uncharacterized ABC transporter permease component YrbE |  | peg.589 |
| Lipoprotein releasing system transmembrane protein LolC |  | peg.543 |
| Outer membrane protein YfgL lipoprotein component of the protein assembly complex (forms a complex with YaeT YfiO and NlpB) |  | peg.2168 |
| Outer membrane lipoprotein carrier protein LolA |  | peg.2408 |
| Outer membrane lipoprotein SmpA a component of the essential YaeT outer-membrane protein assembly complex |  | peg.184 |
| Outer membrane protein assembly factor YaeT precursor |  | peg.712 |
| Lipopolysaccharide ABC transporter ATP-binding protein LptB |  | peg.595 |
| Probable component of the lipoprotein assembly complex (forms a complex with YaeT YfgL and NlpB) |  | peg.3192 |
| Uncharacterized ABC transporter periplasmic component YrbD |  | peg.588 |
| LptA protein essential for LPS transport across the periplasm |  | peg.594 |
| HtrA protease/chaperone protein |  | peg.488 |
| Inner membrane protein YrbG predicted calcium/sodium:proton antiporter |  | peg.1315 |
| Lipoprotein releasing system ATP-binding protein LolD |  | peg.542 |
| Survival protein SurA precursor (Peptidyl-prolyl cis-trans isomerase SurA) | 5.2.1.8 | peg.974 |
| Outer membrane protein NlpB lipoprotein component of the protein assembly complex (forms a complex with YaeT YfiO and YfgL) |  | peg.882 |
| LPS-assembly lipoprotein RlpB precursor (Rare lipoprotein B) |  | peg.1174 |
| Outer membrane protein Imp required for envelope biogenesis |  | peg.973 |
| Uncharacterized ABC transporter auxiliary component YrbC |  | peg.587 |
| Uncharacterized ABC transporter ATP-binding protein YrbF |  | peg.590 |
| **Lipid A-Ara4N pathway ( Polymyxin resistance )** | | |
| **Gene/Enzyme** | **E.C. Number** | **Gene Identification** |
| Polymyxin resistance protein ArnT undecaprenyl phosphate-alpha-L-Ara4N transferase |  | peg.2304 |
| UDP-glucose dehydrogenase | 1.1.1.22 | peg.1923  peg.2755 peg.2888 |
| **KDO2-Lipid A biosynthesis** | | |
| **Gene/Enzyme** | **E.C. Number** | **Gene Identification** |
| UDP-3-O-[3-hydroxymyristoyl] glucosamine N-acyltransferase | 2.3.1.- | peg.713 |
| Arabinose 5-phosphate isomerase | 5.3.1.13 | peg.591 peg.3184 |
| UDP-2 3-diacylglucosamine hydrolase | 3.6.1.- | peg.2870 |
| 3-deoxy-D-manno-octulosonic-acid transferase | 2.-.-.- | peg.333 |
| DNA internalization-related competence protein ComEC/Rec2 |  | peg.540 |
| Lipopolysaccharide ABC transporter ATP-binding protein LptB |  | peg.595 |
| Lipid A biosynthesis lauroyl acyltransferase | 2.3.1.- | peg.2573 peg.2948 |
| 2-Keto-3-deoxy-D-manno-octulosonate-8-phosphate synthase | 2.5.1.55 | peg.762 peg.3185 |
| 3-deoxy-D-manno-octulosonate 8-phosphate phosphatase | 3.1.3.45 | peg.592 |
| LptA protein essential for LPS transport across the periplasm |  | peg.594 |
| UDP-3-O-[3-hydroxymyristoyl] N-acetylglucosamine deacetylase | 3.5.1.- | peg.551 |
| Tetraacyldisaccharide 4&#39;-kinase | 2.7.1.130 | peg.536 |
| Acyl-[acyl-carrier-protein]--UDP-N-acetylglucosamine O-acyltransferase | 2.3.1.129 | peg.715 |
| LPS-assembly lipoprotein RlpB precursor (Rare lipoprotein B) |  | peg.1174 |
| 3-deoxy-manno-octulosonate cytidylyltransferase | 2.7.7.38 | peg.534 peg.3186 |
| Lipid-A-disaccharide synthase | 2.4.1.182 | peg.716 peg.2303 |
| Lipid A export ATP-binding/permease protein MsbA | 3.6.3.25 | peg.537 |
| **LOS core oligosaccharide biosynthesis** | | |
| **Gene/Enzyme** | **E.C. Number** | **Gene Identification** |
| Phosphoheptose isomerase | 5.3.1.- | peg.569 |
| Beta-1 3-glucosyltransferase |  | peg.1675 |
| ADP-L-glycero-D-manno-heptose-6-epimerase | 5.1.3.20 | peg.342 |
| 3-deoxy-D-manno-octulosonic-acid transferase | 2.-.-.- | peg.333 |
| D-glycero-D-manno-heptose 1 7-bisphosphate phosphatase | 3.1.1.- | peg.348 |
| O-antigen ligase |  | peg.2893 |
| ADP-heptose synthase | 2.7.-.- | peg.334 |
| ADP-heptose--lipooligosaccharide heptosyltransferase II | 2.4.1.- | peg.335  peg.338  peg.341 |
| D-glycero-beta-D-manno-heptose 7-phosphate kinase |  | peg.334 |
| **Lipoprotein sorting system** | | |
| **Gene/Enzyme** | **E.C. Number** | **Gene Identıfıcatıon** |
| Lipoprotein releasing system transmembrane protein LolC |  | peg.543 |
| Lipoprotein releasing system ATP-binding protein LolD |  | peg.542 |
| Outer membrane lipoprotein carrier protein LolA |  | peg.2408 |
| **Murein Hydrolases** | | |
| **Gene/Enzyme** | **E.C. Number** | **Gene Identification** |
| Membrane-bound lytic murein transglycosylase D precursor | 3.2.1.- | peg.2833 |
| Beta N-acetyl-glucosaminidase | 3.2.1.52 | peg.3109 |
| N-acetylmuramoyl-L-alanine amidase | 3.5.1.28 | peg.459 peg.1746 |
| D-alanyl-D-alanine carboxypeptidase | 3.4.16.4 | peg.242  peg.689 peg.1139 |
| Soluble lytic murein transglycosylase precursor | 3.2.1.- | peg.1039 |
| Membrane-bound lytic murein transglycosylase B precursor | 3.2.1.- | peg.960 peg.1137 |
| Muramoyltetrapeptide carboxypeptidase | 3.4.17.13 | peg.830 |
| **Peptidoglycan Biosynthesis** | | |
| **Gene/Enzyme** | **E.C. Number** | **Gene Identification** |
| Glucosamine-1-phosphate N-acetyltransferase | 2.3.1.157 | peg.395 |
| UDP-N-acetylmuramoylalanyl-D-glutamate--2 6-diaminopimelate ligase | 6.3.2.13 | peg.562 |
| Monofunctional biosynthetic peptidoglycan transglycosylase | 2.4.2.- | peg.995 |
| D-alanyl-D-alanine carboxypeptidase | 3.4.16.4 | peg.242  peg.689 peg.1139 |
| Cell division protein FtsI [Peptidoglycan synthetase] | 2.4.1.129 | peg.563 |
| UDP-N-acetylenolpyruvoylglucosamine reductase | 1.1.1.158 | peg.532 |
| Glutamine synthetase type I | 6.3.1.2 | peg.2619 |
| Rare lipoprotein A precursor |  | peg.1138 peg.1227 |
| UDP-N-acetylglucosamine 1-carboxyvinyltransferase | 2.5.1.7 | peg.584 |
| Membrane-bound lytic murein transglycosylase B precursor | 3.2.1.- | peg.960 peg.1137 |
| UDP-N-acetylmuramoylalanine--D-glutamate ligase | 6.3.2.9 | peg.559 |
| UDP-N-acetylglucosamine--N-acetylmuramyl-(pentapeptide) pyrophosphoryl-undecaprenol N-acetylglucosamine transferase | 2.4.1.227 | peg.557 |
| Rod shape-determining protein RodA |  | peg.1136 |
| UDP-N-acetylmuramoylalanyl-D-glutamyl-2 6-diaminopimelate--D-alanyl-D-alanine ligase | 6.3.2.10 | peg.561 |
| Glutamate racemase | 5.1.1.3 | peg.2411 |
| UDP-N-acetylmuramate--alanine ligase | 6.3.2.8 | peg.556 |
| Penicillin-binding protein 2 (PBP-2) |  | peg.1135 |
| D-alanine--D-alanine ligase | 6.3.2.4 | peg.555 |
| Phospho-N-acetylmuramoyl-pentapeptide-transferase | 2.7.8.13 | peg.560 |
| N-acetylglucosamine-1-phosphate uridyltransferase | 2.7.7.23 | peg.395 |
| Multimodular transpeptidase-transglycosylase | 2.4.1.129 3.4.-.- | peg.750 |
| **UDP-N-acetylmuramate from Fructose-6-phosphate Biosynthesis** | | |
| **Gene/Enzyme** | **E.C. Number** | **Gene Identification** |
| UDP-N-acetylenolpyruvoylglucosamine reductase | 1.1.1.158 | peg.532 |
| Glucosamine-1-phosphate N-acetyltransferase | 2.3.1.157 | peg.395 |
| Glucosamine--fructose-6-phosphate aminotransferase [isomerizing] | 2.6.1.16 | peg.396 |
| UDP-N-acetylglucosamine 1-carboxyvinyltransferase | 2.5.1.7 | peg.584 |
| Phosphoglucosamine mutase | 5.4.2.10 | peg.164 peg.3164 |
| N-acetylglucosamine-1-phosphate uridyltransferase | 2.7.7.23 | peg.395 |
| **YjeE** | | |
| **Gene/Enzyme** | **E.C. Number** | **Gene Identification** |
| COG3178: Predicted phosphotransferase related to Ser/Thr protein kinases |  | peg.972 |
| ATPase YjeE predicted to have essential role in cell wall biosynthesis |  | peg.1747 |
| Inactive homolog of metal-dependent proteases putative molecular chaperone |  | peg.892 |
| **Recycling of Peptidoglycan Amino Sugars** | | |
| **Gene/Enzyme** | **E.C. Number** | **Gene Identification** |
| Beta N-acetyl-glucosaminidase | 3.2.1.52 | peg.3109 |
| Anhydro-N-acetylmuramic acid kinase | 2.7.1.- | peg.134 |
| UDP-N-acetylmuramate:L-alanyl-gamma-D-glutamyl-meso-diaminopimelate ligase | 6.3.2.- | peg.1125 |
| N-acetylmuramoyl-L-alanine amidase | 3.5.1.28 | peg.459 peg.1746 |
| N-acetylmuramoyl-L-alanine amidase AmpD | 3.5.1.28 | peg.888 |
| AmpG permease |  | peg.468 |
| Aminoacyl-histidine dipeptidase (Peptidase D) | 3.4.13.3 | peg.2271 |
| Muramoyltetrapeptide carboxypeptidase | 3.4.17.13 | peg.830 |
| **Peptidoglycan biosynthesis--gjo** | | |
| **Gene/Enzyme** | **E.C. Number** | **Gene Identification** |
| UDP-N-acetylmuramate:L-alanyl-gamma-D-glutamyl-meso-diaminopimelate ligase | 6.3.2.- | peg.1125 |
| UDP-N-acetylmuramate--alanine ligase | 6.3.2.8 | peg.556 |
| UDP-N-acetylmuramoylalanine--D-glutamate ligase | 6.3.2.9 | peg.559 |
| UDP-N-acetylmuramoylalanyl-D-glutamate--2 6-diaminopimelate ligase | 6.3.2.13 | peg.562 |
| D-alanine--D-alanine ligase | 6.3.2.4 | peg.555 |
| UDP-N-acetylmuramoylalanyl-D-glutamyl-2 6-diaminopimelate--D-alanyl-D-alanine ligase | 6.3.2.10 | peg.561 |
| **Others** | | |
| **Biotin biosynthesis** | | |
| **Gene/Enzyme** | **E.C. Number** | **Gene Identification** |
| Biotin operon repressor |  | peg.3085 |
| Adenosylmethionine-8-amino-7-oxononanoate aminotransferase | 2.6.1.62 | peg.667 peg.1736 |
| Biotin-protein ligase | 6.3.4.15 | peg.3085 |
| 8-amino-7-oxononanoate synthase | 2.3.1.47 | peg.1102 |
| Dethiobiotin synthetase | 6.3.3.3 | peg.1099 |
| Biotin synthase | 2.8.1.6 | peg.1103 |
| Long-chain-fatty-acid--CoA ligase | 6.2.1.3 | peg.111 peg.232 peg.233 peg.1715 peg.2363 peg.3106 |
| 3-ketoacyl-CoA thiolase | 2.3.1.16 | peg.114  peg.153 |
| **Biotin biosynthesis Experimental** | | |
| **Gene/Enzyme** | **E.C. Number** | **Gene Identification** |
| Dethiobiotin synthetase | 6.3.3.3 | peg.1099 |
| Biotin synthase | 2.8.1.6 | peg.1103 |
| Competence protein F homolog phosphoribosyltransferase domain |  | peg.1105 |
| NfuA Fe-S protein maturation |  | peg.2785 |
| Adenosylmethionine-8-amino-7-oxononanoate aminotransferase | 2.6.1.62 | peg.667 peg.1736 |
| 8-amino-7-oxononanoate synthase | 2.3.1.47 | peg.1102 |
| **Thiamin biosynthesis** | | |
| **Gene/Enzyme** | **E.C. Number** | **Gene Identification** |
| Sulfur carrier protein adenylyltransferase ThiF |  | peg.646 |
| Hydroxymethylpyrimidine ABC transporter substrate-binding component |  | peg.2821 |
| Glycine oxidase ThiO | 1.4.3.19 | peg.3189 |
| Thiazole biosynthesis protein ThiG |  | peg.2498 |
| 1-deoxy-D-xylulose 5-phosphate synthase | 2.2.1.7 | peg.288 |
| Phosphomethylpyrimidine kinase | 2.7.4.7 | peg.370  peg.2485 |
| Thiamin ABC transporter substrate-binding component |  | peg.1808 |
| Thiaminase II | 3.5.99.2 | peg.2951 |
| Thiamin biosynthesis protein ThiC |  | peg.2952 |
| Thiamin-phosphate pyrophosphorylase | 2.5.1.3 | peg.239 |
| Thiamin ABC transporter transmembrane component |  | peg.1807 |
| Hydroxyethylthiazole kinase | 2.7.1.50 | peg.238 |
| Sulfur carrier protein ThiS |  | peg.2499 |
| **Menaquinone and Phylloquinone Biosynthesis** | | |
| **Gene/Enzyme** | **E.C. Number** | **Gene Identification** |
| Ubiquinone/menaquinone biosynthesis methyltransferase UbiE | 2.1.1.- | peg.738 |
| **Ubiquinone Biosynthesis** | | |
| **Gene/Enzyme** | **E.C. Number** | **Gene Identification** |
| 2-octaprenyl-6-methoxyphenol hydroxylase | 1.14.13.- | peg.318 |
| Ubiquinone biosynthesis monooxygenase UbiB |  | peg.470  peg.736 |
| Ubiquinone/menaquinone biosynthesis methyltransferase UbiE | 2.1.1.- | peg.738 |
| 3-polyprenyl-4-hydroxybenzoate carboxy-lyase UbiX | 4.1.1.- | peg.1126 |
| Chorismate--pyruvate lyase | 4.1.3.40 | peg.2537 |
| 2-octaprenyl-3-methyl-6-methoxy-1 4-benzoquinol hydroxylase | 1.14.13.- | peg.317  peg.968 |
| NAD(P)H-flavin reductase | 1.5.1.29 1.16.1.3 | peg.725 |
| Protein YigP (COG3165) clustered with ubiquinone biosynthetic genes |  | peg.737 |
| 3-polyprenyl-4-hydroxybenzoate carboxy-lyase | 4.1.1.- | peg.724 |
| **Cobalamin synthesis** | | |
| **Gene/Enzyme** | **E.C. Number** | **Gene Identification** |
| Cobyric acid synthase |  | peg.678 |
| Cobalt-precorrin-6x reductase | 1.3.1.54 | peg.685 |
| Cobalamin biosynthesis protein BluB |  | peg.679 |
| L-threonine 3-O-phosphate decarboxylase | 4.1.1.81 | peg.26 |
| Adenosylcobinamide-phosphate guanylyltransferase | 2.7.7.62 | peg.25 |
| Cobalt-precorrin-8x methylmutase | 5.4.1.2 | peg.688 |
| Cobalamin synthase |  | peg.24 |
| Cobalt-precorrin-2 C20-methyltransferase | 2.1.1.130 | peg.687 |
| Cobalt-precorrin-4 C11-methyltransferase | 2.1.1.133 | peg.682 |
| Cobalt-precorrin-3b C17-methyltransferase |  | peg.686 |
| Nicotinate-nucleotide--dimethylbenzimidazole phosphoribosyltransferase | 2.4.2.21 | peg.28 |
| Cob(I)alamin adenosyltransferase | 2.5.1.17 | peg.680  peg.1026 |
| Adenosylcobinamide-phosphate synthase |  | peg.27 |
| Cobyrinic acid A C-diamide synthase |  | peg.11 |
| **Heme and Siroheme Biosynthesis** | | |
| **Gene/Enzyme** | **E.C. Number** | **Gene Identification** |
| Glutamyl-tRNA synthetase | 6.1.1.17 | peg.2917 |
| Coproporphyrinogen III oxidase aerobic | 1.3.3.3 | peg.2173 |
| Uroporphyrinogen-III synthase | 4.2.1.75 | peg.2467 |
| Glutamate-1-semialdehyde aminotransferase | 5.4.3.8 | peg.369 |
| Porphobilinogen synthase | 4.2.1.24 | peg.826 |
| Radical SAM family enzyme similar to coproporphyrinogen III oxidase oxygen-independent clustered with nucleoside-triphosphatase RdgB |  | peg.359 |
| Coproporphyrinogen III oxidase oxygen-independent | 1.3.99.22 | peg.271 |
| Glutamyl-tRNA reductase | 1.2.1.70 | peg.650 |
| Porphobilinogen deaminase | 2.5.1.61 | peg.2468 |
| Uroporphyrinogen-III methyltransferase | 2.1.1.107 | peg.1226 peg.2009 |
| Ferrochelatase protoheme ferro-lyase | 4.99.1.1 | peg.943 |
| Uroporphyrinogen III decarboxylase | 4.1.1.37 | peg.280 |
| **Coenzyme B12 biosynthesis** | | |
| **Gene/Enzyme** | **E.C. Number** | **Gene Identification** |
| L-threonine 3-O-phosphate decarboxylase | 4.1.1.81 | peg.26 |
| Cobalt-precorrin-6y C5-methyltransferase | 2.1.1.- | peg.684 |
| 5 6-dimethylbenzimidazole synthase flavin destructase family |  | peg.679 |
| Sirohydrochlorin cobaltochelatase | 4.99.1.3 | peg.3026 |
| Cobalt-precorrin-8x methylmutase | 5.4.1.2 | peg.688 |
| Cobalt-precorrin-2 C20-methyltransferase | 2.1.1.130 | peg.687 |
| CobW GTPase involved in cobalt insertion for B12 biosynthesis |  | peg.10 |
| Cobalt-precorrin-6y C15-methyltransferase [decarboxylating] | 2.1.1.- | peg.684 |
| Uroporphyrinogen-III methyltransferase | 2.1.1.107 | peg.1226 peg.2009 |
| Vitamin B12 ABC transporter B12-binding component BtuF |  | peg.20 |
| ChlI component of cobalt chelatase involved in B12 biosynthesis |  | peg.12 |
| Outer membrane vitamin B12 receptor BtuB |  | peg.21 |
| Cobyric acid synthase |  | peg.678 |
| Cobalt-precorrin-6x reductase | 1.3.1.54 | peg.685 |
| Precorrin-6A synthase | 2.1.1.152 | peg.681 |
| Adenosylcobinamide-phosphate guanylyltransferase | 2.7.7.62 | peg.25 |
| Cobalamin synthase |  | peg.24 |
| Cobalt-precorrin-3b C17-methyltransferase |  | peg.686 |
| Cobalt-precorrin-4 C11-methyltransferase | 2.1.1.133 | peg.682 |
| Nicotinate-nucleotide--dimethylbenzimidazole phosphoribosyltransferase | 2.4.2.21 | peg.28 |
| Cob(I)alamin adenosyltransferase | 2.5.1.17 | peg.680 peg.1026 |
| Adenosylcobinamide-phosphate synthase |  | peg.27 |
| Cobalamin biosynthesis protein CobG |  | peg.8 |
| Cobyrinic acid A C-diamide synthase |  | peg.11 |
| CobN component of cobalt chelatase involved in B12 biosynthesis |  | peg.9 |
| **Flavodoxin** | | |
| **Gene/Enzyme** | **E.C. Number** | **Gene Identification** |
| Hypothetical flavoprotein YqcA (clustered with tRNA pseudouridine synthase C) |  | peg.2688 |
| **Pyridoxin (Vitamin B6) Biosynthesis** | | |
| **Gene/Enzyme** | **E.C. Number** | **Gene Identification** |
| D-3-phosphoglycerate dehydrogenase | 1.1.1.95 | peg.292  peg.1598  peg.2737  peg.3179 |
| 4-hydroxythreonine-4-phosphate dehydrogenase | 1.1.1.262 | peg.975 |
| Pyridoxamine 5&#39;-phosphate oxidase | 1.4.3.5 | peg.2799 |
| Erythronate-4-phosphate dehydrogenase | 1.1.1.290 | peg.2421 |
| Pyridoxine 5&#39;-phosphate synthase | 2.6.99.2 | peg.481 |
| D-erythrose-4-phosphate dehydrogenase | 1.2.1.72 | peg.2649 |
| Phosphoserine aminotransferase | 2.6.1.52 | peg.3121 |
| 1-deoxy-D-xylulose 5-phosphate synthase | 2.2.1.7 | peg.288 |
| NAD-dependent glyceraldehyde-3-phosphate dehydrogenase | 1.2.1.12 | peg.1726 peg.2458 |
| **NAD and NADP cofactor biosynthesis global** | | |
| **Gene/Enzyme** | **E.C. Number** | **Gene Identification** |
| Quinolinate synthetase | 2.5.1.72 | peg.873 |
| Nicotinate phosphoribosyltransferase | 2.4.2.11 | peg.2071 |
| Quinolinate phosphoribosyltransferase [decarboxylating] | 2.4.2.19 | peg.887 |
| NAD kinase | 2.7.1.23 | peg.46 |
| ADP-ribose pyrophosphatase | 3.6.1.13 | peg.1771 peg.2731 |
| NAD synthetase | 6.3.1.5 | peg.3191 |
| Tryptophan 2 3-dioxygenase | 1.13.11.11 | peg.2122 |
| Kynurenine formamidase | 3.5.1.9 | peg.2352 |
| Nicotinamide-nucleotide adenylyltransferase NadM family | 2.7.7.1 | peg.2731 |
| Glutamine amidotransferase chain of NAD synthetase |  | peg.87  peg.3191 |
| L-aspartate oxidase | 1.4.3.16 | peg.1407 |
| Nicotinate-nucleotide adenylyltransferase | 2.7.7.18 | peg.1132 |
| Kynureninase | 3.7.1.3 | peg.2353 |
| Nicotinamidase | 3.5.1.19 | peg.1597 |
| **YgfZ** | | |
| **Gene/Enzyme** | **E.C. Number** | **Gene Identification** |
| Quinolinate synthetase | 2.5.1.72 | peg.873 |
| Aminodeoxychorismate lyase | 4.1.3.38 | peg.518 |
| Glutathione synthetase | 6.3.2.3 | peg.308 |
| Dihydropteroate synthase | 2.5.1.15 | peg.165 |
| Chorismate synthase | 4.2.3.5 | peg.1056 |
| YgfY COG2938 |  | peg.1409 |
| Flavodoxin reductases (ferredoxin-NADPH reductases) family 1 |  | peg.406  peg.1182  peg.2938 |
| Thiol:disulfide interchange protein DsbC |  | peg.3006 |
| Iron-sulfur cluster assembly ATPase protein SufC |  | peg.78 |
| Molybdenum cofactor biosynthesis protein MoaA |  | peg.3095 |
| Aspartate carbamoyltransferase | 2.1.3.2 | peg.304 |
| Twin-arginine translocation protein TatA |  | peg.734 |
| Phosphoribosylformylglycinamidine cyclo-ligase | 6.3.3.1 | peg.1254 |
| Dihydroorotate dehydrogenase | 1.3.3.1 | peg.2416 |
| Dihydroorotase | 3.5.2.3 | peg.2881 |
| Lipoate synthase |  | peg.1142 |
| Aminomethyltransferase (glycine cleavage system T protein) | 2.1.2.10 | peg.2696 |
| S-formylglutathione hydrolase | 3.1.2.12 | peg.993 |
| GTP cyclohydrolase II | 3.5.4.25 | peg.282  peg.2560 |
| S-(hydroxymethyl)glutathione dehydrogenase | 1.1.1.284 | peg.992 |
| GTPase and tRNA-U34 5-formylation enzyme TrmE |  | peg.356 |
| Adenosylcobinamide-phosphate synthase |  | peg.27 |
| Folate-dependent protein for Fe/S cluster synthesis/repair in oxidative stress |  | peg.2723 |
| FIG001341: Probable Fe(2+)-trafficking protein YggX |  | peg.3218 |
| Ribose-phosphate pyrophosphokinase | 2.7.6.1 | peg.654 |
| Gamma-glutamyl phosphate reductase | 1.2.1.41 | peg.1131 |
| Rhodanese-related sulfurtransferase |  | peg.1825 |
| Aconitate hydratase | 4.2.1.3 | peg.244  peg.245  peg.691  peg.3152 |
| Iron binding protein IscA for iron-sulfur cluster assembly |  | peg.2160 |
| Succinate dehydrogenase iron-sulfur protein | 1.3.99.1 | peg.2912 |
| Queuosine Biosynthesis QueC ATPase |  | peg.1304 |
| Biotin synthase | 2.8.1.6 | peg.1103 |
| Aconitate hydratase 2 | 4.2.1.3 | peg.1297 |
| Cysteine desulfurase | 2.8.1.7 | peg.2159 |
| Phosphoribosyl-AMP cyclohydrolase | 3.5.4.19 | peg.2779 |
| Orotate phosphoribosyltransferase | 2.4.2.10 | peg.2521 |
| Glutamate--cysteine ligase | 6.3.2.2 | peg.1795 |
| Phosphoglycerate kinase | 2.7.2.3 | peg.2648 |
| Amidophosphoribosyltransferase | 2.4.2.14 | peg.1203 |
| tRNA-i(6)A37 methylthiotransferase |  | peg.1064 |
| Coproporphyrinogen III oxidase oxygen-independent | 1.3.99.22 | peg.271 |
| Ferric uptake regulation protein FUR |  | peg.183 |
| Glutamate synthase [NADPH] small chain | 1.4.1.13 | peg.760 |
| L-aspartate oxidase | 1.4.3.16 | peg.1407 |
| Cysteine desulfurase SufS subfamily | 2.8.1.7 | peg.76 |
| 2-amino-4-hydroxy-6-hydroxymethyldihydropteridine pyrophosphokinase | 2.7.6.3 | peg.150  peg.981 |
| Fumarate hydratase class I aerobic | 4.2.1.2 | peg.2728 |
