# Supplementary material for: Genomic analysis reveals the biotechnological and industrial potential of levan producing halophilic extremophile, Halomonas smyrnensis AAD6T
Source: Springerplus. 2015 Aug 4;4:393. doi: 10.1186/s40064-015-1184-3 (PMC4523562; doi:10.1186/s40064-015-1184-3)
Supplement: Supplementary file 7 — Additional file 7: Text document including the additional information on other cellular processes represented in H. smyrnensis AAD6T genome. [file 40064_2015_1184_MOESM7_ESM.docx]

**SpringerPlus**

**Genomic analysis reveals the biotechnological and industrial potential of levan producing halophilic extremophile, *Halomonas smyrnensis* AAD6T**

**Elif Sogutcu^1^, Tugba Ozer^1^, Muzaffer Arikan^2^, Zeliha Emrence^2^, Ebru Toksoy Oner^1^, Duran Ustek^3^, Kazim Yalcin Arga^1,§^**

^1^ Department of Bioengineering, Marmara University, Goztepe 34722, Istanbul, Turkey

^2^ Department of Genetics, Institute for Experimental Medicine, Istanbul University, Capa 34093, Istanbul, Turkey

^3^ Department of Medical Genetics, School of Medicine, REMER, Medipol University, 34810, Istanbul, Turkey

^§^Corresponding author:

Assoc. Prof. Dr. Kazim Yalcin Arga

E-mail: kazim.arga@marmara.edu.tr

**Additional information on other cellular processes** **represented in *H. smyrnensis* AAD6T genome**

**Cell Division**

The genome annotation results pointed out fifteen genes, which encode proteins associated with cell division. A significant part of these proteins belonged to the family of Fts and results showed that *H. smyrnensis* AAD6T has typical components of cell division (Supplementary Figure S5). Divisome assembly is originated by polymerization of the widely conserved tubulin homologue FtsZ into a ring-like structure, which is placed at the midcell with at least ten additional proteins, the majority of which are likely to be involved in remodeling the peptidoglycan cell wall at the division site. ZipA is enlisted to the assembling of Z-ring via a direct interaction with FtsZ after the initiation of cell division [1]. FtsA, which is an actin superfamily member, shows ATPase activity, stabilizes Z-rings at the membrane, and recruits downstream components. FtsK functions as a chromosome segregator, which remove proteins from DNA as it translocates and is also required for the targeting of FtsQ, FtsL and FtsI to the septum [2-4]. FtsQ, FtsL and FtsB are broadly conserved proteins of cell division, and are located in the pathway between the assembled Z-ring scaffold, consisting of FtsZ and FtsZ binding proteins, and the septal peptidoglycan synthesis machinery [2]. FtsW, a SEDS (shape, elongation, division and sporulation) family member, transports lipid-linked peptidoglycan precursors from the inner to the outer leaflet of the cytoplasmic membrane. It is required for localization of FtsI and also plays a role in the stabilization of the Z-ring during cell division [5-6]. Besides these Fts genes, a septum formation protein Maf, sporulation initiation inhibitor protein Soj, stage 0 sporulation protein J and chromosome (plasmid) partitioning proteins ParA and ParB were also identified.

*H. smyrnensis* AAD6T presents a Min system, consisting of three proteins MinCDE, which controls the division site selection. The executive inhibitory protein MinC blocks the formation of polar Z-ring polymer, interacting with a peripheral membrane ATPase and the oscillator MinD to become membrane-associated and activated [7]. MinE drives a process that imparts topological determinant by the periodic oscillation of MinC and MinD from pole to pole [8-10]. In addition, the genome possesses SlmA protein that is required for nucleoid occlusion and inhibition of Z-ring assembly close to the nucleoid.

The rod-shape of the microorganism is provided by functioning of four rod-shape determining proteins: RodA, MreB, MreC and MreD, which in turn serves as a genetic support to the observations.

**Transcription Process**

The genome annotation identified several transcription process associated genes, which show high similarities to corresponding orthologous of *C. salexigens*. These include genes encoding the α, ß and Ω subunits of RNA polymerase together with the ß and ß’ subunit genes, nine ORFs encoding bacterial transcription factors, three transcription termination proteins (NusA, NusB and Rho), two transcription elongation factors (GreA and GreB), and one each of transcription anti-termination protein (NusG), transcriptional activator (RfaH), transcription-repair coupling factor and transcription accessory protein (S1 RNA-binding domain). Furthermore, five transcription initiation genes (sigma factors RpoD, RpoE, RpoH, RpoN and RpoS), which promote the attachment of RNA polymerase to specific initiation sites and are then released, and two anti-sigma factors that bind to sigma factors and inhibit their transcriptional activity for RseA and RseB proteins were identified. Only one sigma factor (RpoN), which is responsible for the expression of enzymes involved in arginine catabolism, belongs to the Sigma-54 factor family. The σ70 family includes primary sigma factors as well as related alternative sigma factors; hence, six σ70 factors of seven might not be surprising. There are also two regulatory proteins NsrR and IscR, member of the Rrf2 family of transcription repressors. NsrR protein is specifically dedicated to sensing nitric oxide in a variety of pathogenic and non-pathogenic bacteria [11] and IscR protein is responsible as a repressor of the iscRSUA operon, and *in vitro* transcription reactions have revealed that IscR has a repressive effect on the IscR promoter in the case of iron-sulfur cluster loading [12].

**Lipopolysaccharides**

The lipopolysaccharides (LPSs) are complex amphiphilic macromolecules embedded in the outer leaflet of the external membrane of gram-negative bacteria and they are indispensable for the bacterial growth, viability, and for the correct assembly [13]. As a gram-negative bacterium, H. *smyrnensis* AAD6T possesses LPSs in the external leaflet of its outer membrane. LPSs are contributing greatly to the structural integrity of the bacteria, help to resist antimicrobial compounds and environmental stresses, and are involved in many aspects of host–bacterium interactions in the case of extremophile bacteria, survival under harsh conditions [14,15]. Several genes associated with lipopolysaccharide assembly were identified in *H. smyrnensis* AAD6T. “Arabinose 5-phosphate isomerase (EC 5.3.1.13)” is one of these and it is a key enzyme in the biosynthesis of LPSs. Also some transporter proteins were found such as YrbC, YrbD, YrbE, YrbF, LolA, LolC, LolD, LptA and LptB, and several outer membrane proteins like SmpA, YaeT, Imp, NlpB and YfgL, related to LPS biosynthesis.

**Quorum Sensing**

Many gram-negative bacteria produce signal molecules such as N-acyl homoserine lactones (AHLs) which take part in cell-density-dependent gene-expression mechanism known as ‘quorum sensing’ [16-19]. Several cellular functions including the production of EPS in *Pantoea stewartii* [20] and in *Sinorhizobium melitoti* [21] were regulated by the cell-to-cell communication. Some members of the moderately halophilic genus *Halomonas*, such as *H. eurihalina*, *H. maura*, *H. ventosae* and *H. anticariensis* produce EPS and auto-inducer molecules that are involved in the cell-to-cell signaling process [16, 18]. In levan producer *H. smyrnensis* AAD6 genome, several genes such as LuxS, LuxP, RpoS, RpoN (σ^54^) that play roles in bacterial cell-to-cell communication were identified [19, 22].

**Arsenic Resistance**

Arsenic (As) ions are frequently present as environmental pollutants and microbial activity is known to play a key role in their bioavailability [23]. Though they are very toxic for most forms of life, some bacteria have evolved different strategies to survive arsenic stress. These arsenic-resistant bacteria are widely distributed in nature so that they have been isolated from both arsenic-rich and arsenic-free environments [24]. Arsenic resistance is mediated either by its selective removal through methylation, by an energy requiring detoxification process catalyzed by the arsenate reductase protein (ArsC), where arsenate, As (V), is first reduced to arsenite, As (III), and then effluxed, or by dissimilatory reduction of As (V) to obtain energy for growth [25].

The *H. smyrnensis* AAD6T genome also carries multiple genes that are potentially involved in arsenic resistance, as has also been reported for *Halomonas* *sp.* strain HAL1 [26], *Halomonas* *sp.* strain GFAJ-1 [27], and *Halomonas sp*. strain KM-1 [28]. Genes for “Arsenate reductase (EC 1.20.4.1)”, “Arsenic resistance protein, ArsH”, “Arsenic efflux pump protein”, “Arsenical resistance operon repressor” and “Transcriptional regulator, ArsR family” were present within the draft genome. Presence of these genes suggests that *H. smyrnensis* AAD6 is an arsenite-specific expulsion prokaryote rather than a dissimilatory arsenic-reducing prokaryote [25]. The predicted arsenic resistance genes in the genome, as well as scientific reports for arsenic resistance of several *Halomonas* species support the genomic potential of arsenic resistance of *H. smyrnensis* AAD6T and put it as a candidate organism for bioremediation studies.

**Supplementary references**

1. Adams DW and Errington J. Bacterial cell division: assembly, maintenance and disassembly of the Z ring. Nat Rev Microbiol. 2009; 7: 642-653.
2. Goehring NW and Beckwith J. Diverse Paths to Midcell: Assembly of the Bacterial Cell Division Machinery. Curr Biol. 2005; 15: 514-526.
3. Graham JE, Sivanathan V, Sherratt DJ, Arciszewska LK. FtsK translocation on DNA stops at XerCD-dif. Nucleic Acids Res. 2010; 38: 72-81.
4. Chen JC, Beckwith J. FtsQ, FtsL and FtsI require FtsK, but not FtsN, for co-localization with FtsZ during *Escherichia coli* cell division. Mol Microbiol. 2001; 42: 395-413.
5. Boyle DS, Khattar MM, Addinall SG, Lutkenhaus J, Donachie WD. FtsW is an essential cell-division gene in *Escherichia coli*. Mol Microbiol. 1997; 24: 1263-1273.
6. Mohammadi T, Van Dam V, Sijbrandi R, Vernet T, Zapun A, Bouhss A, et al. Identification of FtsW as a transporter of lipid-linked cell wall precursors across the membrane. EMBO J. 2011; 30: 1425-1432.
7. Hu Z, Mukherjee A, Pichoff S, Lutkenhaus J. The MinC component of the division site selection system in *Escherichia coli* interacts with FtsZ to prevent polymerization. Proc Natl Acad Sci USA. 1999; 96: 14819–14824.
8. Raskin DM, de Boer PA. The MinE ring: an FtsZ-independent cell structure required for selection of the correct division site in *E. coli*. Cell. 1997; 91: 685–694.
9. Hu Z, Lutkenhaus J. Topological regulation of cell division in *Escherichia coli* involves rapid pole to pole oscillation of the division inhibitor MinC under the control of MinD and MinE. Mol Microbiol. 1999; 34: 82–90.
10. Raskin DM, de Boer PA. MinDE-dependent pole-to-pole oscillation of division inhibitor MinC in *Escherichia coli*. J Bacteriol. 1999; 181: 6419–6424.
11. Tucker NP, Hicks MG, Clarke TA, Crack JC, Chandra G, Le Brun NE, et al. The transcriptional repressor protein NsrR senses nitric oxide directly via a [2Fe-2S] cluster. PLoS ONE. 2008; 3: e3623.
12. Schwartz CJ, Giel JL, Patschkowski T, Luther C, Ruzicka FJ, Beinert H, et al. IscR, an Fe-S cluster-containing transcription factor, represses expression of *Escherichia coli* genes encoding Fe-S cluster assembly proteins. Proc Natl Acad Sci USA. 2001; 98: 14895-14900.
13. Raetz C, Whitfield C. Lipopolysaccharide Endotoxins. Annu Rev Biochem. 2002; 71: 635-700.
14. Pieretti G, Nicolaus B, Poli A, Corsaro MM, Lanzetta R, Parrilli M. Structural determination of the O-chain polysaccharide from the haloalkaliphilic *Halomonas alkaliantarctica* bacterium strain CRSS. Carbohydrate Research. 2009; 344: 2051–2055.
15. Silipo A, Gargiulo V, Sturiale L, Marchetti R, Prizeman P, Grant WD, et al. The structure of the carbohydrate backbone of the lipooligosaccharide from an alkaliphilic *Halomonas* sp. Carbohydrate research. 2010; 13: 1971-1975.
16. [Tahrioui](http://mic.sgmjournals.org/search?author1=Ali+Tahrioui&sortspec=date&submit=Submit) A, Quesada E, Llamas I. The hanR/hanI quorum-sensing system of *Halomonas anticariensis*, a moderately halophilic bacterium. Microbiology. 2011; 157, 3378-3387.
17. Eberl L. Quorum sensing in the genus *Burkholderia*. [Int J of Med Microbiol](http://www.sciencedirect.com/science/journal/14384221). 2006; 296: 103-110.
18. [Llamas I](http://www.ncbi.nlm.nih.gov/pubmed?term=Llamas%20I%5BAuthor%5D&cauthor=true&cauthor_uid=15909077), [Quesada E](http://www.ncbi.nlm.nih.gov/pubmed?term=Quesada%20E%5BAuthor%5D&cauthor=true&cauthor_uid=15909077), [Martínez-Cánovas MJ](http://www.ncbi.nlm.nih.gov/pubmed?term=Mart%C3%ADnez-C%C3%A1novas%20MJ%5BAuthor%5D&cauthor=true&cauthor_uid=15909077), [Gronquist M](http://www.ncbi.nlm.nih.gov/pubmed?term=Gronquist%20M%5BAuthor%5D&cauthor=true&cauthor_uid=15909077), [Eberhard A](http://www.ncbi.nlm.nih.gov/pubmed?term=Eberhard%20A%5BAuthor%5D&cauthor=true&cauthor_uid=15909077), [González JE](http://www.ncbi.nlm.nih.gov/pubmed?term=Gonz%C3%A1lez%20JE%5BAuthor%5D&cauthor=true&cauthor_uid=15909077). Quorum sensing in halophilic bacteria: detection of N-acyl-homoserine lactones in the exopolysaccharide-producing species of *Halomonas.* Extremophiles. 2005; 9: 333-341.
19. González JE, Keshavan N. Messing with bacterial quorum sensing. Microbiol Mol Biol Rev. 2006; 70: 859–875.
20. BeckVon Bodman S, Majerczak DR, Coplin DL. A negative regulator mediates quorum-sensing control of exopolysaccharide production in *Pantoea stewartii* subsp. Stewartii. [Proc Natl Acad Sci](http://www.google.com.tr/url?sa=t&rct=j&q=&esrc=s&source=web&cd=1&cad=rja&ved=0CCEQFjAA&url=http%3A%2F%2Fwww.pnas.org%2F&ei=EemsUJSgJM_NswbnjoHQAw&usg=AFQjCNF8L8b8kaHKmCj0CPzwGkSLYL9tsA&sig2=ottFnI3bB9iKr31bv4NolA) USA. 1998; 95: 7687-7692.
21. Marketon MM, Glenn SA, Eberhard A, Gonza´lez JE. Quorum sensing controls exopolysaccharide production in *Sinorhizobium meliloti*. J Bacteriol. 2003; 185: 325-331.
22. Ng WL, Bassler BL. Bacterial quorum-sensing network architectures. Annu Rev Genet. 2009; 43: 197–222.
23. Cervantes C, Ji G, Ramírez JL, Silver S. Resistance to arsenic compounds in microorganisms. FEMS Microbiol Rev. 1994; 15: 355-67.
24. Takeuchi M, Kawahata H, Gupta LP, Kita N, Morishita Y, Ono Y, et al. Arsenic resistance and removal by marine and non-marine bacteria. J Biotechnol. 2007; 127: 434-442.
25. Lloyd JR. Microbial transformation of Arsenic in Aquifers Biological Chemistry of Arsenic. In Biological Chemistry of Arsenic, Antimony and Bismuth*.* Edited by Sun H. Wiley. 2011. p. 135-142.
26. Lin Y, Fan H, Hao X, Johnstone L, Hu Y, Wei G, et al. Draft genome sequence of *Halomonas* sp. strain HAL1, a moderately halophilic arsenite-oxidizing bacterium isolated from goldmine soil. J Bacteriol. 2012; 194: 199-200.
27. Wolfe-Simon F, Switzer Blum J, Kulp TR, Gordon GW, Hoeft SE, Pett-Ridge J, et al. A bacterium that can grow by using arsenic instead of phosphorus. Science. 2011; 332: 1163-1166.
28. Kawata Y, Kawasaki K, Shigeri Y. Draft Genome Sequence of *Halomonas* *sp*. Strain KM-1, a Moderately Halophilic Bacterium That Produces the Bioplastic Poly(3-Hydroxybutyrate). J Bacteriol. 2012; 194: 2738.
